# Rhamnolipids and Essential Oils: A New Approach to Managing Bacteria Associated with Skin Diseases

**DOI:** 10.3390/microorganisms14102254

**Published:** 2026-10-04

**Authors:** Lucas Araujo Leite, Maria Olimpia Pereira Sereia, Gabriel Barbieri Mestieri, Raphael Culim Neves, Aline Martins dos Santos, Suzana Gonçalves Carvalho, Carolina Rosai Mendes, Marlus Chorilli, Guilherme Dilarri, Jonas Contiero

**Affiliations:** 1 Department of General and Applied Biology, Institute of Biosciences, São Paulo State University (UNESP), Rio Claro 13506-900, SP, Brazil; la.leite@unesp.br (L.A.L.); raphael.culim@unesp.br (R.C.N.); 2 School of Pharmaceutical Sciences, São Paulo State University (UNESP), Araraquara 14800-901, SP, Brazilmarlus.chorilli@unesp.br (M.C.); 3 Department of Biological Sciences, Santa Catarina State University (UDESC), Laguna 88790-000, SC, Brazil; carolina.rosai@unesp.br; 4 Institute for Research in Bioenergy, São Paulo State University (UNESP), Rio Claro 13500-230, SP, Brazil

**Keywords:** biosurfactant, rhamnolipid, essential oils, *Cutibacterium acnes*, *Staphylococcus aureus*, antimicrobial film, sodium alginate

## Abstract

The rise of antimicrobial-resistant pathogens has increased interest in biologically derived compounds for treating dermal infections. This study evaluates the effect of rhamnolipid, a biosurfactant produced by *Pseudomonas aeruginosa*, in combination with essential oils of oregano (*Origanum vulgare*), tea tree (*Melaleuca alternifolia*), and cinnamon (*Cinnamomum cassia*) against *Cutibacterium acnes* and *Staphylococcus aureus*, with or without incorporation into a sodium alginate-based film. Minimum bactericidal concentrations (MBC), biofilm inhibition development, and time-kill assays were used to assess antimicrobial activity. Results indicate MBCs of 800 mg/L for rhamnolipid, 0.05% for oregano, 0.2% for tea tree oil, and 0.5% for cinnamon against *C. acnes*. For *S. aureus*, the MBCs are 2500 mg/L, 0.07%, 0.3%, and 0.5%, respectively. When combined the rhamnolipids and the essential oils, the MBC for *C. acnes* were reduced to 500 mg/L of rhamnolipid and essential oils of oregano, tea tree and cinnamon were reduced to 0.03%, 0.1% and 0.3%, respectively, and for *S. aureus* were reduced to 1500 mg/L of rhamnolipid and essential oils of oregano, tea tree and cinnamon were reduced to 0.05%, 01% and 0.3%, respectively. A sodium alginate-based polymer film was formulated with rhamnolipid, maintaining antimicrobial efficacy and water-retention capacity but with reduced mechanical strength. These findings suggest a promising topical application for treating dermal infections.

## 1. Introduction

Over the years, contaminants and pathogenic bacteria have become increasingly resistant to conventional treatment methods, necessitating ongoing investment in new prevention and therapy strategies. Among the greatest challenges is the bacterial biofilm—an extracellular matrix secreted by several bacterial species—which grants enhanced resistance to antimicrobial agents, promotes cellular aggregation, and facilitates rapid growth and colonization of various environments [1].

Two bacterial species, *Cutibacterium acnes* and *Staphylococcus aureus*, have exhibited a significant rise in infection rates and notable antibiotic resistance [2]. Although both are commensal residents of the human skin microbiota, they can act as opportunistic pathogens. These bacteria are frequently implicated in dermal infections and, more recently, have been detected in infections associated with surgical implants, including joint prostheses, silicone-based devices, and cardiac implants [3,4].

Biosurfactants, specifically glycolipids, have low critical micelle concentrations (CMC), greater biodegradability, and reduced aquatic toxicity than synthetic surfactants, while maintaining structural and interfacial functionality under extreme salinity, temperature, and pH conditions [1]. Rhamnolipids are glycolipid biosurfactants composed of a fatty acid chain (hydrophobic region) and a sugar moiety (hydrophilic region). They are widely used in the cosmetic industry to formulate creams, shampoos, and deodorants because they emulsify phases, reduce surface tension, exert antimicrobial and bactericidal activity, and show high skin compatibility with minimal allergenic effects [5,6]. Rhamnolipid is primarily produced by *Pseudomonas aeruginosa*, and its antimicrobial action against Gram-positive bacteria is well documented. This activity is attributed to its amphipathic nature, which allows it to interact with bacterial membrane lipid bilayers, altering membrane fluidity and permeability or disrupting membrane structure and causing cellular damage [7].

Because of its diverse applications, this metabolite is used across sectors including enhanced oil recovery (EOR), environmental bioremediation, cosmetics, and sustainable agriculture. On the other hand, the cost of large-scale production is an obstacle, preventing its competitiveness with those of petrochemical origin. Studies to reduce the cost of obtaining this resource are underway, and it is believed that this input can be obtained sustainably in the future, thereby contributing to the bioeconomy [5].

Essential oils are volatile oils or plant-derived essences characterized by resistance to hydrolysis [8]. They comprise a complex mixture of volatile compounds, including terpenes, aldehydes, alcohols, and esters. These compounds naturally serve to attract pollinators and act as antifungal and antibacterial agents. In human applications, essential oils have been used for their antioxidant, antiseptic, anti-inflammatory properties, and in food preservation [8,9]. Initially used in perfumery and food preservation, their effectiveness against antibiotic-resistant bacteria—such as methicillin-resistant *Staphylococcus aureus*—has sparked renewed interest in their application as alternatives to synthetic antimicrobial agents [10].

Biopolymer films are widely utilized in the cosmetic and medical industries to treat dermal infections. They isolate the affected area, maintain skin hydration, and ensure prolonged contact between bioactive compounds and the infected site, which enhances therapeutic efficacy. Moreover, research on the development and application of biologically derived films has increased significantly, driven by the demand for biodegradable and biocompatible products. In the food industry, such films are already applied as edible coatings to extend shelf life and prevent microbial contamination [11]. These same materials are gaining relevance in the medical field, particularly as supportive structures for wound healing and tissue regeneration [12]. Nowadays, alginate-sodium base films are widely used in these areas because they are non-toxic, biodegradable, and biocompatible. They are also readily obtained from brown seaweeds [13].

In this context, the present study aims to evaluate the antimicrobial effects and efficacy of combining rhamnolipid with essential oils, with or without incorporation, in sodium alginate-based films against *Cutibacterium acnes* and *Staphylococcus aureus*.

## 2. Materials and Methods

### 2.1. Rhamnolipid Production

Rhamnolipid production, extraction, and recovery were carried out following the methodology described by Yoshimura et al. (2022) [14].

### 2.2. Microorganisms

The microorganism used for rhamnolipid production was *Pseudomonas aeruginosa* LBI 2A1, a random mutant strain obtained from the Laboratory of Industrial Microbiology (LMI) at UNESP —Rio Claro, SP, Brazil. The strain is preserved in an ultra-low temperature freezer at −80 °C in cryovials containing lysogeny broth supplemented with 20% glycerol.

The bacteria utilized for antimicrobial activity assays were *Cutibacterium acnes* (ATCC 6919) and *Staphylococcus aureus* (ATCC 25923). *C. acnes* strain was cultured in Reinforced Clostridial Medium (RCM) at 37 °C, under aerobic conditions for 24 h, while *S. aureus* strain was cultured in Luria–Bertani (LB) broth, also at 37 °C, under aerobic conditions for 24 h. Both strains were maintained on agar slants and subcultured before use in assays.

### 2.3. Essential Oils

The essential oils used in this study were oregano essential oil (*Origanum vulgare*) (OEO), cinnamon essential oil (*Cinnamomum cassia*) (CEO), and tea tree essential oil (*Melaleuca alternifolia*) (TTO). All essential oils were obtained from Ferquima Indústrias e Comércio Ltda — Vargem Grande Paulistana, SP, Brazil. Each essential oil has a 98% purity degree.

The chemical compositions of the oils are described as follows:

- Oregano essential oil (*Origanum vulgare*): β-Caryophyllene (3.24%), Carvacrol (72.15%), γ-Terpinene (4.61%), Linalool (2.74%), *p*-Cymene (4.88%), Thymol (2.48%).
- Tea tree essential oil (*Melaleuca alternifolia*): Terpinen-4-ol (41%), γ-Terpinene (21%), α-Terpinene (9%), α-Terpineol (4%), α-Pinene (3%), 1,8-Cineole (1%).
- Cinnamon essential oil (*Cinnamomum cassia*): Cinnamyl alcohol (0.24%), Cinnamaldehyde (83.23%), Benzaldehyde (0.85%), Coumarin (0.98%).

### 2.4. Emulsion Preparation

For emulsion preparation, the analyzed concentrations of rhamnolipid and essential oils were solubilized in sterile distilled water and stirred using an agitator at maximum speed for 4 min.

### 2.5. Determination of Minimum Bactericidal Concentration (MBC)

Bacterial strains were reactivated in their respective growth media. Next, 1 mL of bacterial culture was transferred to each experimental flask containing the appropriate culture medium and the tested compound at varying concentrations. The treatments included a control flask without any compounds, as well as flasks containing different concentrations of RL (rhamnolipid), OEO (oregano essential oil), TTO (tea tree oil), and CEO (cinnamon essential oil). The cultures were then incubated for 24 h at 37 °C. After incubation, a 100 µL aliquot was collected from each sample and serially diluted sixfold, yielding a final dilution of 10^−6^. The diluted samples were inoculated onto respective solid media using the spread plate technique with a Drigalski spatula and incubated for an additional 24 h. Colony counts were performed thereafter. The MBC was defined as the lowest concentration of each compound that leads to a ≥99% reduction in viable bacterial counts compared to the untreated control, determined by colony-forming units (CFU). The experiment was carried out with three independent replicates.

### 2.6. Biofilm Inhibition Assay

The 96-well microplates were prepared by adding 100 µL of each compound at its respective MBC concentration to individual wells, followed by 100 µL of bacterial inoculum standardized at 1 × 10^5^ CFU/mL, resulting in a final volume of 200 µL per well. A control row containing medium without any antimicrobial agents was used as a negative control. The plate was sealed with paraffin and incubated at 37 °C for 24 h under static conditions to allow biofilm formation on the well surfaces.

After incubation, wells were washed three times with sterile distilled water to remove planktonic cells. The plate was then dried in an oven at 60 °C for 45 min. Subsequently, 200 µL of 1% (*w*/*v*) crystal violet solution was added to each well and incubated at room temperature for 15 min. Remove excess dye and unbound residues by washing the wells three additional times with sterile water. The retained biofilm was then solubilized with 150 µL of 30% acetic acid.

Finally, 100 µL of the dissolved biofilm solution from each well was transferred to cuvettes, and optical density was measured at 590 nm (OD_590_) using a UV-Vis spectrophotometer. Biofilm inhibition percentage was calculated using the following equation [15]:

(1) $$\mathrm{Innibity}\;\mathrm{porcentage}\left(\%\right)=\;\frac{\mathrm{ODcontrol}-\mathrm{ODSample}}{\mathrm{ODcontrol}}\times \;100$$

The assay was performed in technical triplicate with three independent experiments.

### 2.7. Time-Kill Assay

The time-kill assay was performed based on the guidelines outlined in document M26-A from the Clinical & Laboratory Standards Institute (CLSI). Analyses were conducted in technical triplicate and in two independent experiments. Test tubes were prepared containing the MBC of each compound, along with a control tube containing no antimicrobial agents. Each tube was inoculated with bacterial cells in their respective culture media.

The tubes were incubated at 37 °C under aerobic and static conditions, and 100 µL aliquots were collected at 0, 1, 2, 3, and 4 h. After each time point, serial dilutions were performed in sterile saline solution, followed by plating on solid culture media using the spread plate technique. Petri dishes were incubated under optimal conditions for each bacterial strain to allow colony formation and enumeration.

Based on the minimum dilution and plated volume used for viable cell enumeration, the limit of detection (LOD) was 1 × 10^5^ CFU/mL, corresponding to 5 log_10_ CFU/mL. This method was later used to evaluate the interaction effects between rhamnolipid and essential oils [16].

### 2.8. Fluorescence Microscopy Assay

*Cutibacterium acnes* cultures at a concentration of 10^5^ CFU mL^−1^ were exposed to rhamnolipid at its previously determined MBC for 1 h. Following exposure, the bacterial cells were centrifuged at 6000× *g* for 7 min. The supernatant was discarded, and the pellet was resuspended in 1 mL of saline solution (0.86% NaCl) to dilute the compound and halt its bactericidal activity. Bacterial cells were then stained with 10 μg × mL^−1^ propidium iodide (PI) (Sigma-Aldrich, Darmstadt, Germany) and 20 μg × mL^−1^ 4′,6-diamidino-2-phenylindole (DAPI) (Sigma-Aldrich, Darmstadt, Germany). DAPI naturally penetrates all cells through the cytoplasmic membrane and stains them green under fluorescence, while PI selectively penetrates cells with compromised membranes, staining them red [17]. This dual staining allows for the visualization of membrane integrity and the detection of membrane disruption caused by the compound. The positive control (PC) for membrane damage was generated by thermal stress, while the negative control (NC) consisted of untreated cells [18]. Numerical Aperture used was 100 ×/0.125 ∞/0.17 oil and field of view (FN) of 25 mm and the resolution was 5440 × 3648.

Additionally, the potential role of rhamnolipid in interfering with chromosome segregation during bacterial cell division was investigated using a *Bacillus subtilis spo0J-gfp* fusion mutant strain. These cells were cultured in casein hydrolysate medium supplemented with 20μgmL^−1^ tryptophan to induce expression of the Spo0J-GFP fusion protein [19]. After reaching 10^5^ CFU × mL^−1^, cultures were treated with rhamnolipid at its MBC for 15 min. The cells were then centrifuged at 6000× *g* for 7 min, and the pellet was resuspended in 1 mL of 0.86% NaCl solution for microscopic analysis. The negative control consisted of mutant cells not exposed to any treatment.

All samples were immobilized on agarose-coated microscope slides and analyzed using an Opticam O600R fluorescence microscope (Doral, FL, USA) equipped with a 12.3 MP monochromatic camera (Opticam, Doral, FL, USA). Image acquisition and processing were performed using OPTHD software version M4.1 (Opticam, Doral, FL, USA). For data analysis and quantification, a total of 100 cells (n = 100) per treatment group were evaluated. All microscopy experiments were performed with three independent replicates.

### 2.9. Preparation of Sodium Alginate Film

To evaluate the applicability of the antimicrobial compounds, a pharmaceutical film was developed. The film was prepared using sodium alginate by the casting method [13]. The formulation consisted of 2% (*w*/*v*) sodium alginate and 10% (*w*/*w*, relative to alginate) glycerol. Distilled water was added, and the mixture was stirred for one hour. After homogenization, the solution was poured into a Petri dish and dried in a forced-air oven at 40 °C for 18 h [13].

For the formulation of the film containing rhamnolipid and essential oils, the same procedure was followed, with rhamnolipid and essential oils added at their MBC concentration after the initial one hour of stirring. The solution was then stirred for an additional 10 min before casting.

### 2.10. Films Characterization

The thickness of the films was analyzed in triplicate by a digital micrometer MDC-Lite (Mitutoyo^®^) (Kawasaki, Japan) at five random points on the sections.

Mechanical properties of the films were measured using the Universal Texture Analyzer TA-XT2 equipment (Stable Micro Systems, Godalming, UK), using a spherical probe (5 mm). The probe descent speed was 1 mm × s^−1^, and this speed was kept constant at 0.1 mm s^−1^ throughout the test. The trigger force was 0.049 N. Force versus displacement curves were recorded by the Exponent Connect Lite software version 6 until the moment of membrane rupture and used to determine the following parameters:

(2) $$\mathrm{Ps}=\frac{F}{A}$$

where Ps = puncture strength; *F* = force (N) required to rupture the film; *A* = membrane cross-sectional area (cm^2^) [*A* = 2rd, where r is the radius of the hole and d is the thickness of the film].

(3) $$\mathrm{Eb}\;(\%)=\frac{\sqrt{r^{2}+d^{2}}-r}{r}\;\times \;100$$

where Eb = elongation at break; r = exposed radius at the plate hole; d = displacement of the device from the point of contact to film rupture.

(4) $$\mathrm{Ep}=\frac{\mathrm{AUC}}{V}$$

where Ep = perforation energy; AUC = area under the curve (force versus displacement); *V* = membrane volume located in the plate hole.

### 2.11. Liquid Uptake Profile

An adapted Enslin apparatus was used to study the liquid absorption profile of the films in a phosphate buffer pH 7.4. The films were cut into pieces of 1 cm^2^ accurately weighed and placed on the sintered glass filter of the funnel. The liquid absorption profile was measured through the volume absorbed at time intervals (1, 10, 60, and 120 min) relative to the initial mass of the sample. Analyzes were performed in triplicate.

### 2.12. Water Vapor Permeability (WVP)

Water vapor permeability (WVP) was carried out using the gravimetric method, for this purpose, circular sections of the membranes were weighed and placed on top of glass flasks (11 mm opening) containing 10 mL of distilled water (relative humidity 100%). Films were also fixed in empty (water-free) vials as a reference. Both were previously weighed and placed inside a desiccator containing silica gel (relative humidity 0%). At specific intervals (0, 24, 48, 120, and 168 h), the mass loss profile was obtained for each sample and the WVP was calculated using the following equation:

(5) $$\mathrm{WVP}=\frac{G{}^{\circ}\times L}{Ae\times Po\times (\mathrm{RH}1-\mathrm{RH}2)}$$

where WVP = permeability to water vapor (g × mm)/(m^2^ × dia × kPa); G° = rate of mass gain (g/day); L = initial membrane thickness (mm); Ae = exposed membrane area (m^2^); Po = saturation pressure of water vapor at the test temperature (KPa); (UR1 − UR2) = relative humidity gradient.

### 2.13. In Vitro Bioadhesion

The bioadhesive strength of the films was evaluated by measuring using a TA.XT2 texture analyzer plus, operating in adhesive test mode. Mucin discs (mucin-type II, Sigma-Aldrich^®^, Burlington, MA, USA), previously hydrated with phosphate buffer pH 7.4. The films were attached to a cylindrical probe with double-sided adhesive tape. The probe was then lowered to 0.5 N until the mucin discs and film were in contact, which was maintained for 60 s. Then, the probe was lifted at a constant speed of 0.5 mm × s^−1^, until the two surfaces were completely separated. The force (N) and the maximum work for each sample.

### 2.14. Antimicrobial Assay of the Sodium Alginate Film

To evaluate the antimicrobial activity of the sodium alginate film incorporated with rhamnolipid, the agar diffusion method was employed [16]. Three film discs (1 cm in diameter) were cut and placed onto Petri dishes containing Reinforced Clostridial Medium (RCM). *Cutibacterium acnes* was previously cultured to reach a concentration of 1.5 × 10^8^ CFUmL^−1^. From this culture, an aliquot was serially diluted four times using 0.85% (*w*/*v*) saline solution. A 20 µL sample of the final dilution was spread onto RCM agar plates using a Drigalski spatula.

After inoculation, three 1 cm diameter discs of sodium alginate film containing rhamnolipid at its MBC were placed onto the agar surface. The plates were then incubated at 37 °C for 24 h. Following incubation, inhibition of bacterial growth in the area surrounding each disc was visually evaluated.

Plates containing only sodium alginate film discs without rhamnolipid were used as a negative control. The experiment was performed in two independent replicates.

## 3. Results

### 3.1. Minimal Bactericidal Concentration (MBC)

The results for *C. acnes* were promising, as the tested compounds demonstrated low concentrations that reduced viable bacterial growth by ≥99% (Table 1). These findings align with previous literature on the antimicrobial activity of essential oils, except for oregano essential oil (OEO), which performed even better than reported in earlier studies [8,20,21,22].

For *S. aureus*, higher concentrations of the compounds were required, as expected given its known antibiotic resistance, including resistance to methicillin (Table 2). However, the essential oils showed only a slight increase in MBCs compared with *C. acnes*. Notably, oregano essential oil presented a lower MBC than those found in the literature [10].

The application of rhamnolipid–essential oil emulsions significantly reduced the MBCs required to inhibit both bacterial strains’ growth (Table 3 and Table 4). For *C. acnes*, the effective combination consisted of 500 mg/L of rhamnolipid with 0.03%, 0.1%, and 0.3% of OEO, TTO, and CEO, respectively. For *S. aureus*, the MBCs were achieved with 1500 mg/L of rhamnolipid combined with 0.05%, 0.1%, and 0.3% of OEO, TTO, and CEO, respectively.

The variation in the MBC values among the essential oils is attributed to their chemical composition. The antimicrobial potential of essential oils is mainly conferred by compounds from the terpene family [23]. OEO, for instance, contains approximately 72% carvacrol, a monoterpenoid with strong antimicrobial properties, making it the most effective among the tested oils. In contrast, CEO lacks terpenes and is composed mainly of cinnamaldehyde, a phenylpropene with antimicrobial activity, although less potent and slower-acting than terpenes [24]. TTO exhibited moderate antimicrobial activity due to its composition, which includes 41% terpinen-4-ol.

Rhamnolipid functions as a surfactant, disrupting the integrity of microbial membranes. It increases membrane fluidity and permeability, which leads to the leakage of cytoplasmic contents and ultimately results in cell death. Furthermore, its amphiphilic nature facilitates the diffusion of essential oils through both the medium and bacterial membranes, enhancing their antimicrobial effectiveness [25]. Moreover, the emulsification of rhamnolipid with essential oils contributes to the stabilization of volatile components, prolonging their presence in the environment and enhancing antimicrobial activity [26].

### 3.2. Biofilm Inhibition Development

**Figure 1:**
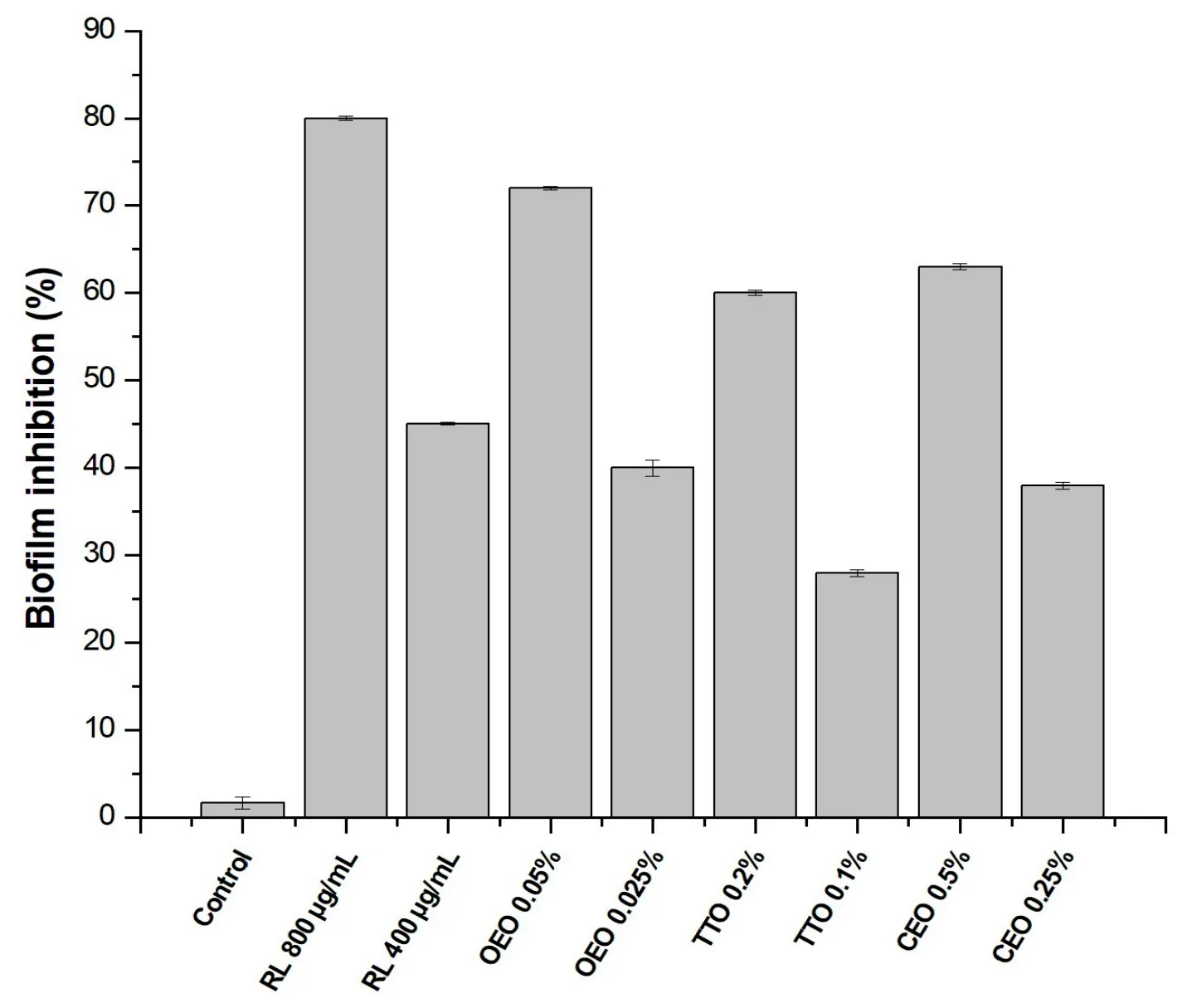
Percentage of biofilm inhibition of *C. acnes* by isolated compounds.

**Figure 2:**
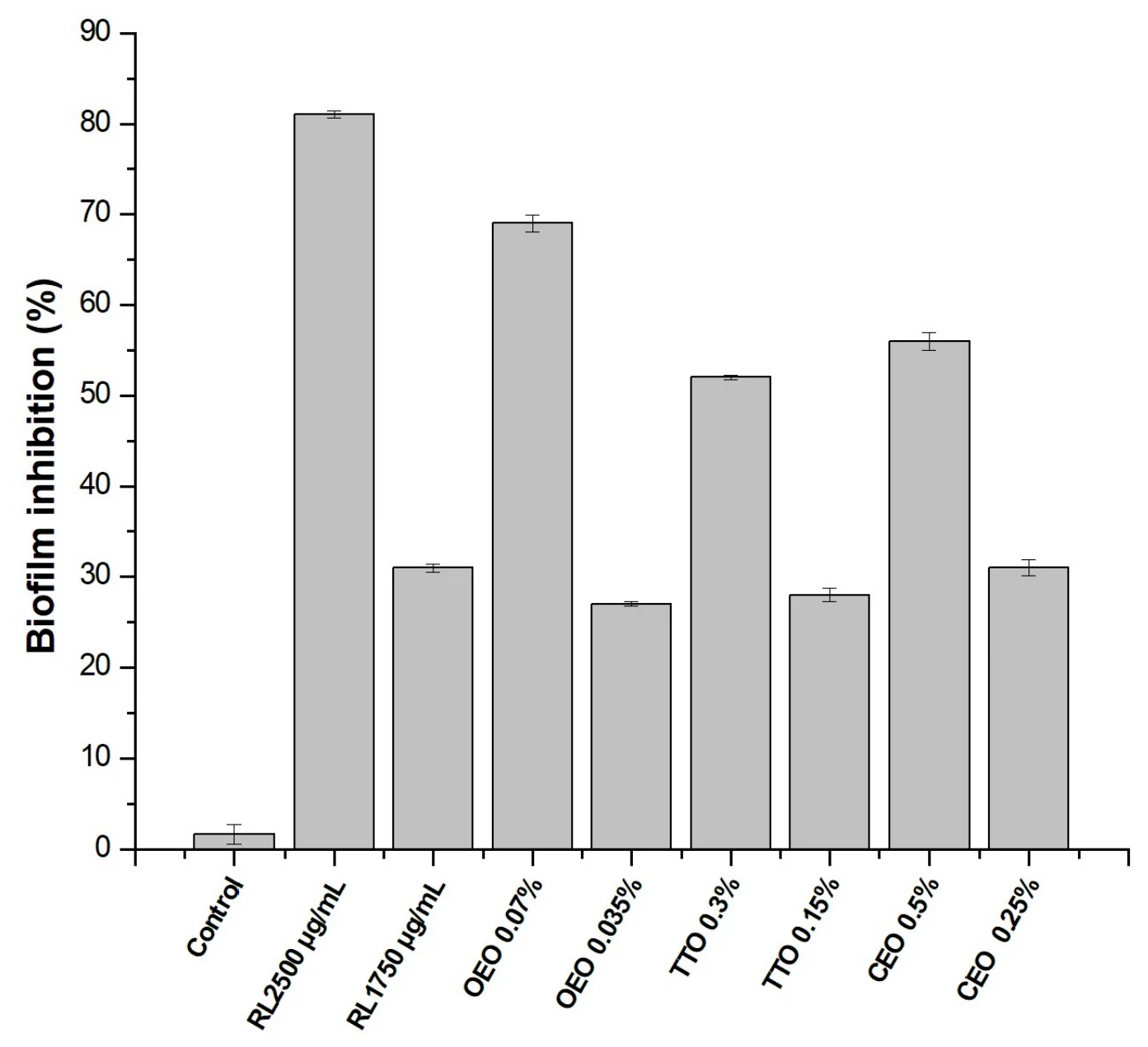
Percentage of biofilm inhibition of *S*. *aureus* by isolated compounds.

When the MBC of the tested compounds was applied to the bacterial strains, their effectiveness in inhibiting biofilm formation was evaluated (Figure 1 and Figure 2). Rhamnolipid showed a stronger inhibitory effect on biofilm formation than the essential oils. However, the essential oils, especially OEO, also demonstrated a significant ability to prevent biofilm formation. This effect can probably be attributed to the high concentration of carvacrol, a monoterpenoid phenol present in OEO, which is known to interfere with biofilm precursor pathways and structural stability [10]. Even though the MBC varied for each compound, the inhibition profiles were similar across both *C. acnes* and *S. aureus* strains.

Among the tested agents, rhamnolipid achieved the highest biofilm inhibition rate (~80%), followed by OEO with ~70% inhibition. These results underscore the potential of both agents, individually and in combination, to prevent bacterial biofilms in dermal infections.

Combining rhamnolipid and essential oils enhanced biofilm inhibition, even at reduced concentrations of both agents. The emulsified formulation achieved over 50% biofilm reduction, highlighting the enhanced activity interaction between the biosurfactant and the extracellular matrix.

**Figure 3:**
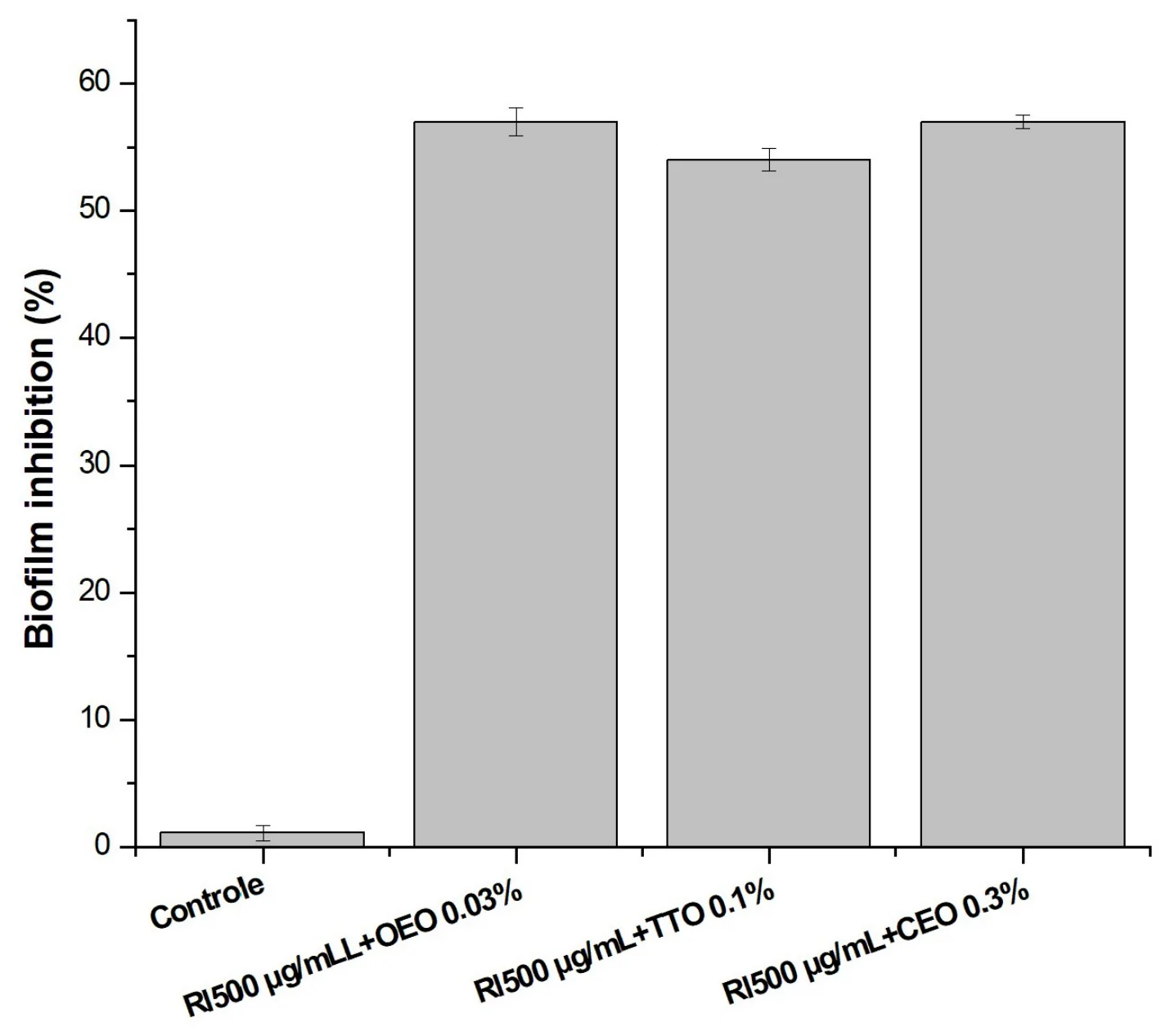
Percentage of biofilm inhibition of *C. acnes* by emulsified compounds.

**Figure 4:**
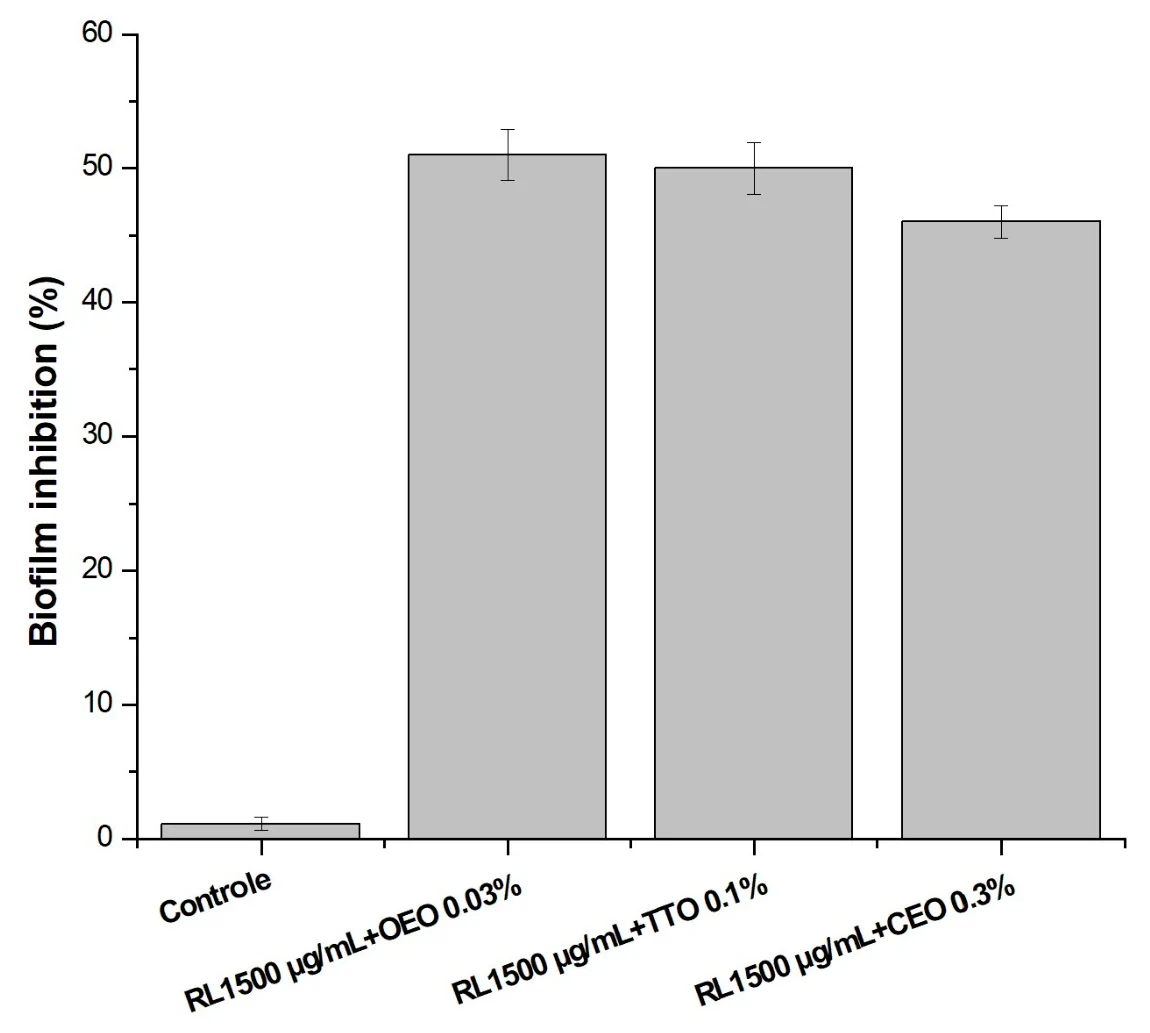
Percentage of biofilm inhibition of *S. aureus* by emulsified compounds.

Due to the nature of the emulsion, rhamnolipid facilitates deeper penetration and destabilization of the biofilm matrix, resulting in a greater inhibitory effect than either compound used individually. This enhanced efficacy is illustrated in Figure 3 and Figure 4.

### 3.3. Time-Kill Assay

**Figure 5:**
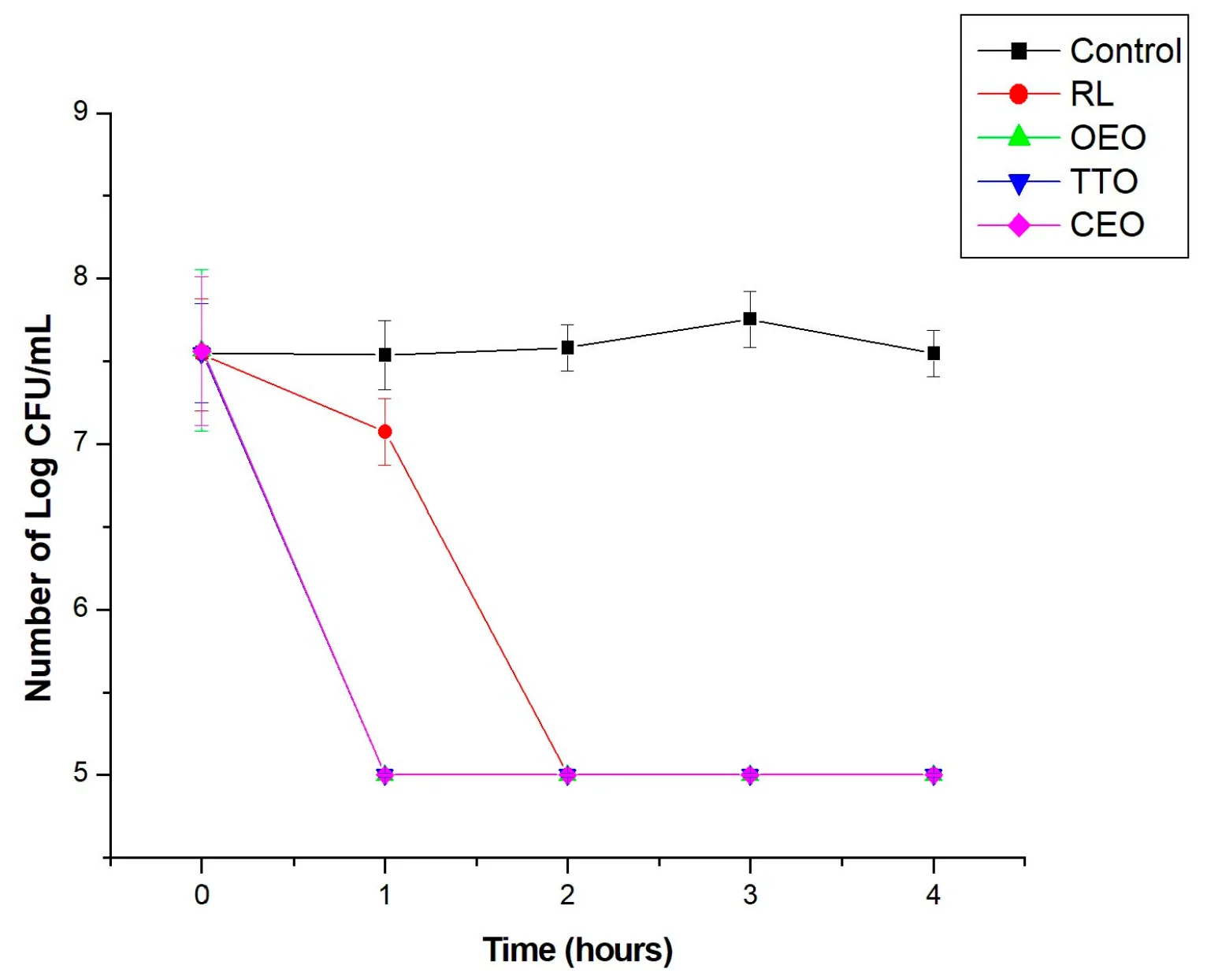
Results of the time-kill assay for *C. acnes* with isolated compounds.

**Figure 6:**
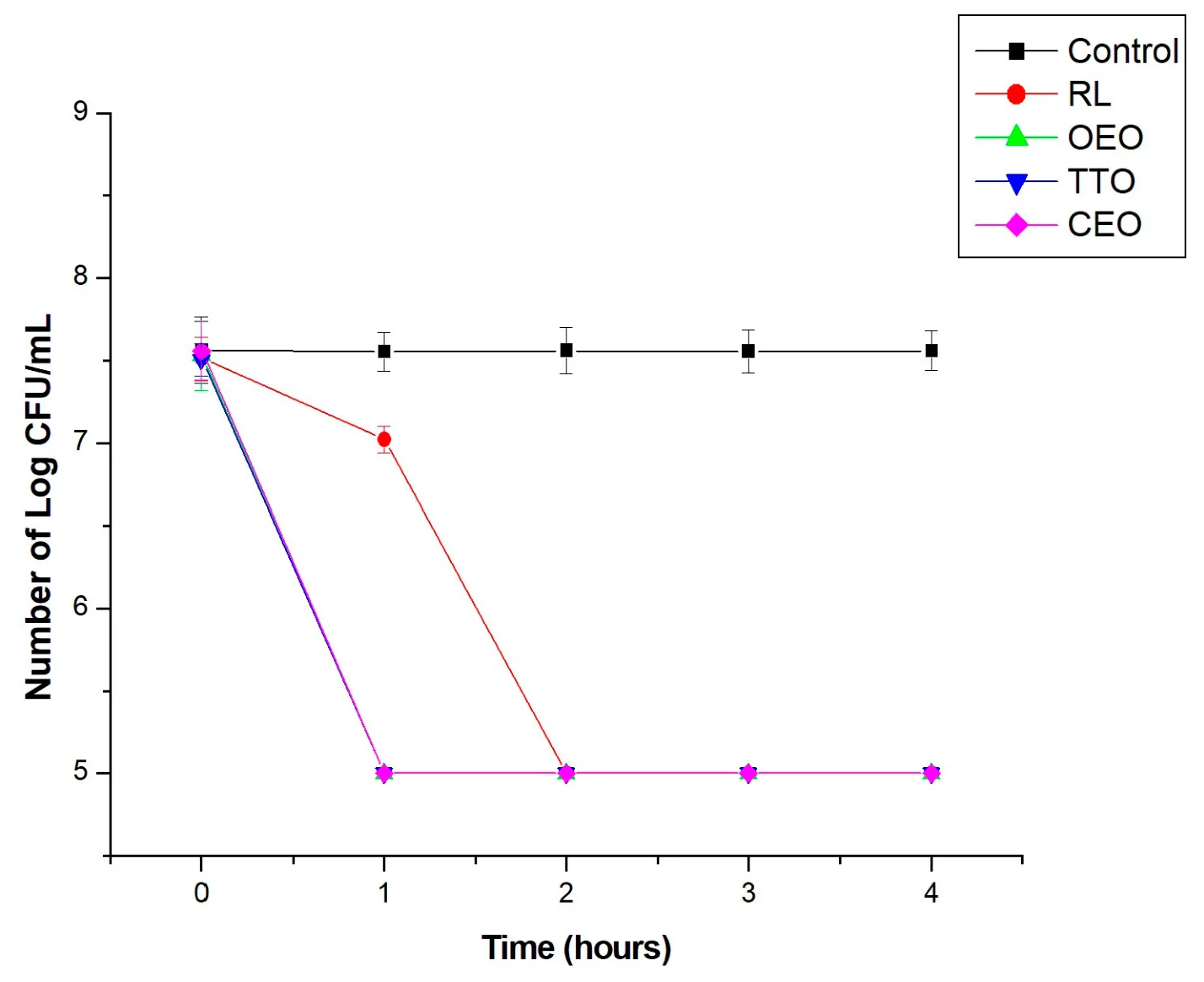
Results of the time-kill assay for *S. aureus* with isolated compounds.

**Figure 7:**
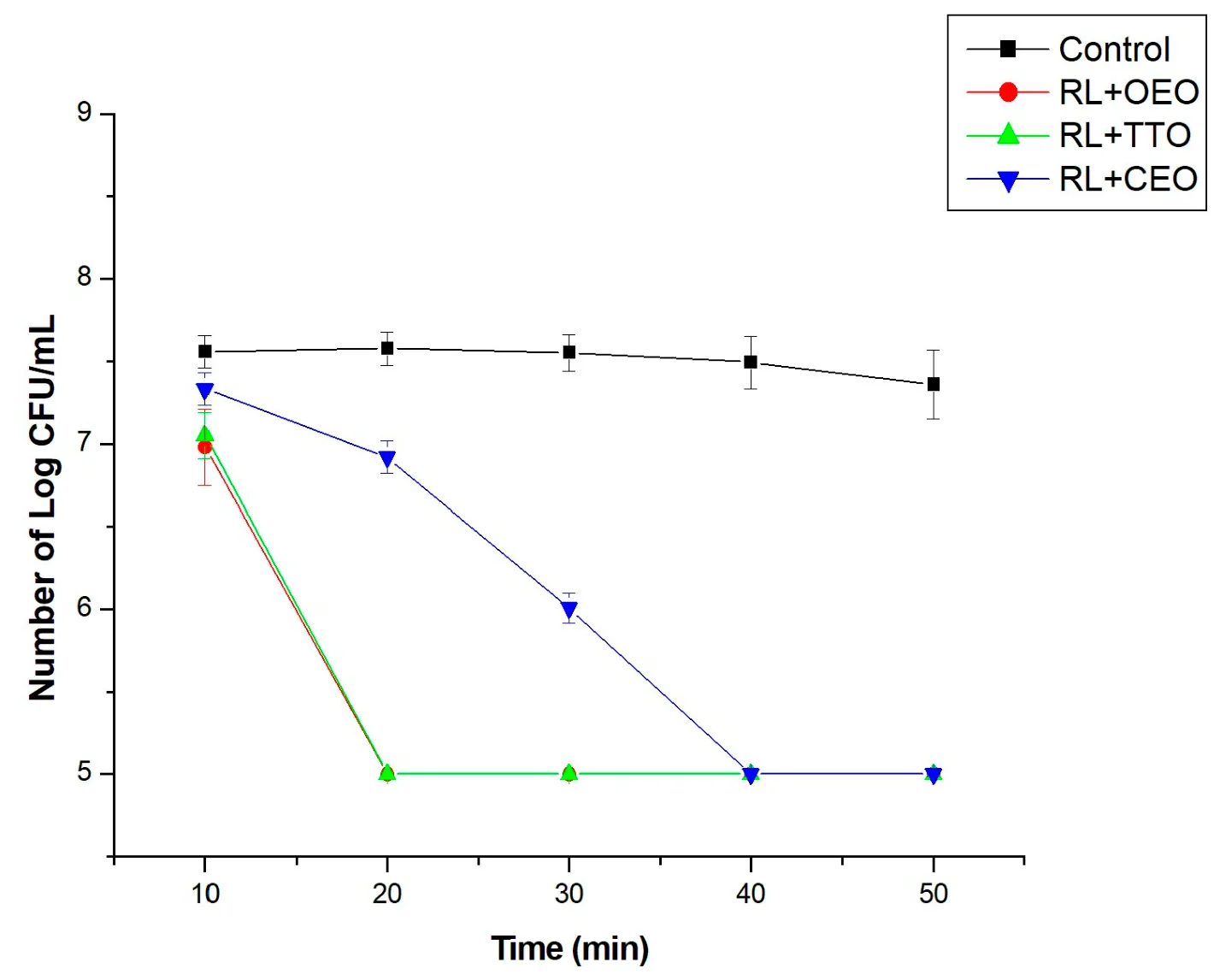
Results of the time-kill assay for *C. acnes* using emulsified compounds.

**Figure 8:**
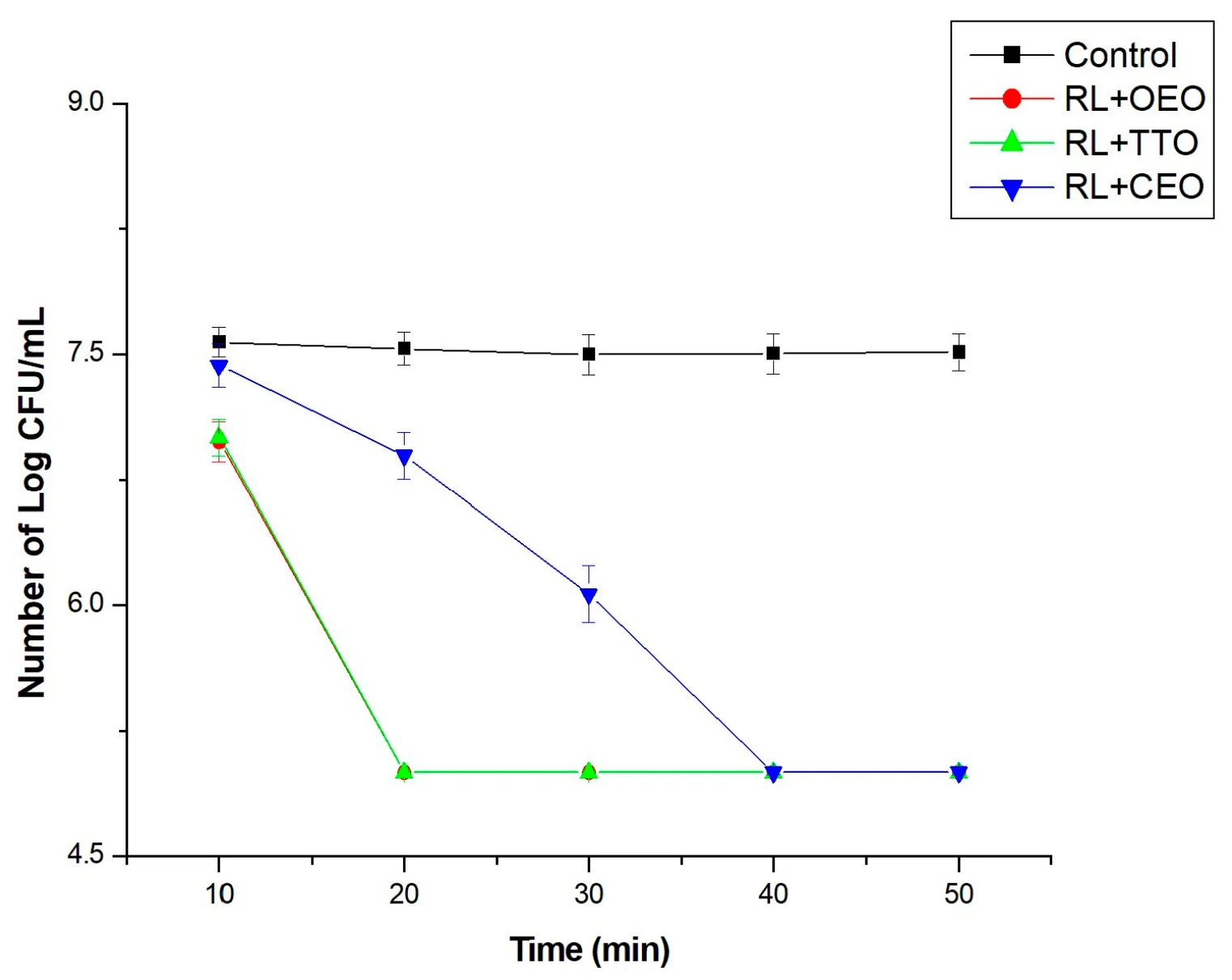
Results of the time-kill assay for *S. aureus* using emulsified compounds.

Time-kill assays revealed that rhamnolipid reduced the bacterial population by half within the first hour and reached the LOD of both *C. acnes* and *S. aureus* colonies after two hours of exposure (Figure 5 and Figure 6). In contrast, the essential oils of oregano, tea tree, and cinnamon reduced viable bacterial colonies after only one hour of exposure for both strains. Emulsified formulations demonstrated even greater efficacy, with total bacterial inhibition occurring within minutes (Figure 7 and Figure 8).

These findings confirmed the significant bactericidal effects of both rhamnolipid and essential oils against *C. acnes* and *S. aureus*, although their action profiles varied. Rhamnolipid reduced approximately 65% of the bacterial population within one hour and reached below the assay detection limit within two hours. These results align with previous findings that reported strong antimicrobial activity of rhamnolipid in topical formulations against *C. acnes*, with low minimum inhibitory concentrations [27].

Rhamnolipids act by incorporating into bacterial membranes, destabilizing the lipid bilayer and promoting cell lysis. Additionally, as a biosurfactant, it can enhance the penetration of hydrophobic compounds into microbial cells, further supporting its lethality. In contrast, essential oils exhibited a nearly instantaneous bactericidal effect. The LOD was reached within the first hour of exposure for both bacterial species. This rapid action is consistent with the known antimicrobial properties of essential oils [27,28]. Oregano essential oil is rich in carvacrol and thymol, which are phenolic compounds that disrupt cell membranes, leading to rapid cell death.

Similarly, tea tree oil, which contains high levels of terpinen-4-ol, decreased the bacterial count below the assay detection limit in one hour (<5 log_10_ CFU/mL). This compound is known to impair cell membranes and disrupt ion transport and respiration with high antimicrobial activity in volatile essential oil compounds, including terpinen-4-ol [28].

Cinnamon essential oil produced similar results within one hour, although research indicates its effectiveness may depend somewhat on the concentration used. The primary component of cinnamon oil, cinnamaldehyde, effectively inhibits biofilm formation and bacterial enzyme activity, even against multidrug-resistant pathogens. Although the action time was similar to the other essential oils, it is possible that under suboptimal conditions, cinnamon oil might require a slightly longer period to exert its effect due to the slower intracellular penetration and metabolism inhibition [29].

The faster action of essential oils than rhamnolipid alone can be attributed to the high reactivity of their phenolic and aldehydic structures, which readily interact with proteins and lipids in the bacterial membrane. Nonetheless, rhamnolipid’s slower but sustained effect could be beneficial in controlled-release systems or prolonged topical treatments [30].

Importantly, emulsified formulations of rhamnolipid and essential oils outperformed the individual compounds (Figure 7 and Figure 8). The rhamnolipid–oregano oil emulsion, for example, exhibited a rapid and effective bactericidal effect, decreasing the bacterial growth in both species within just 20 min of exposure. A similar outcome was observed for the rhamnolipid–tea tree oil emulsion, which also reached the LOD within 20 min. In contrast, the rhamnolipid–cinnamon oil emulsion demonstrated slightly slower antimicrobial activity, achieving full inhibition of *C. acnes* and *S. aureus* after 30 min of exposure.

These results highlight the strong antimicrobial potential of all emulsified combinations, with the oregano and tea tree oil formulations showing the fastest bactericidal activity. Rhamnolipid may enhance the dispersion and penetration of essential oils into bacterial cells, increasing their efficacy.

Moreover, combining rhamnolipid with essential oils produced more stable emulsions with lower volatility. These characteristics support controlled, prolonged release of active compounds, ultimately reducing the time required for antimicrobial action compared with their isolated application [26].

### 3.4. Fluorescence Microscopy Assays

Membrane integrity studies of *C. acnes* cells after exposure to rhamnolipid were performed using fluorescence microscopy assays. Cells were analyzed after 30 min of exposure to rhamnolipid at its bactericidal concentration. Red and green fluorescence filters were used to visualize propidium iodide (PI) and DAPI staining in the bacterial cells. DAPI is typically visualized using a blue filter because its fluorescence emission peaks between 461 and 465 nm. Nonetheless, DAPI can also be viewed with a green filter, as it has a broad emission peak that extends into the green region (around 500 nm), particularly when it binds to DNA [31,32]. Since our microscope is equipped only with green and red filters (green/GFP and red/Texas Red), the DAPI signal in our microscopy images appears green. Therefore, cells with intact membranes appeared green, as PI is unable to penetrate the cytoplasmic membrane [17]. In contrast, cells with damaged membranes were stained red, as the damage caused by rhamnolipid allowed PI to enter.

**Figure 9:**
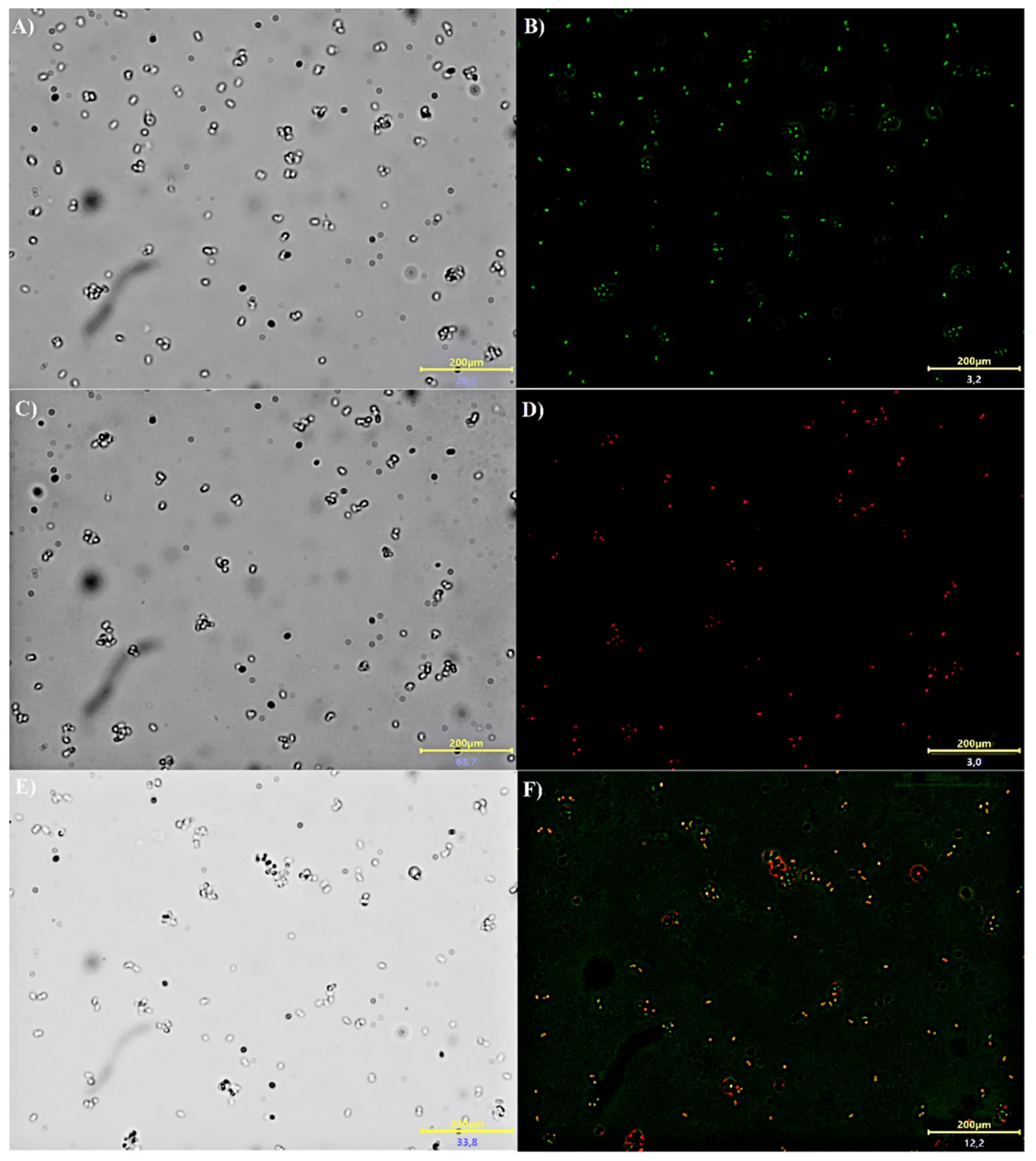
Fluorescence microscopy of *C. acnes* with 800 µg/mL of rhamnolipids. (**A**) Negative control with phase contrast. (**B**) Negative control with fluorescence with green and red filters. (**C**) Positive control with phase contrast. (**D**) Positive control with fluorescence with green and red filters. (**E**) *C. acnes* cells after 1 h of contact with rhamnolipids with phase contrast. (**F**) *C. acnes* cells after 1 h of contact with rhamnolipids with fluorescence with green and red filters. The scale bar corresponds to 200 μm; magnification of ×100.

**Figure 10:**
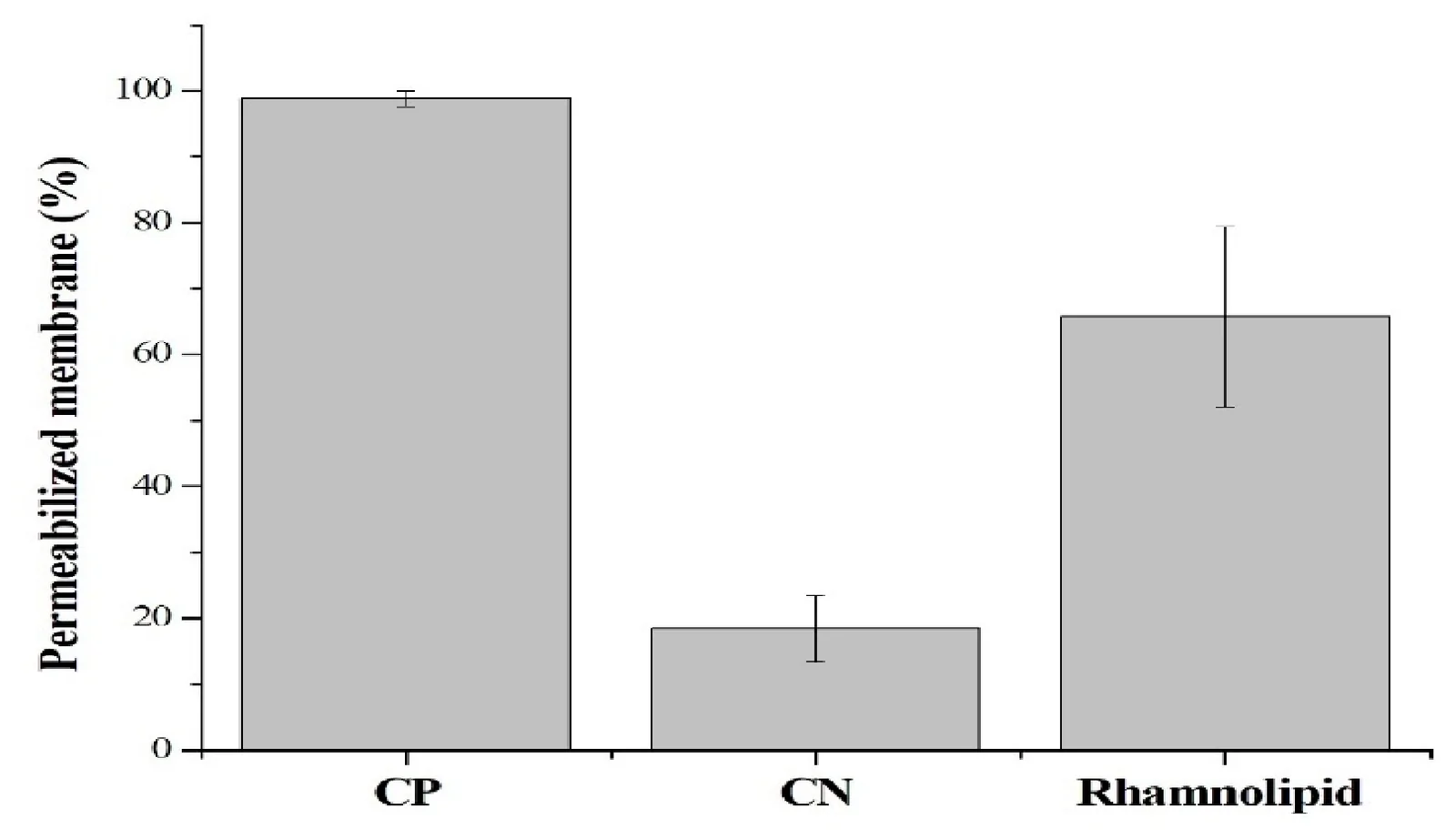
Graphic of the percentage of cells with permeabilized membranes in the fluorescence microscopy assays showing the positive control, negative control and the rhamnolipid. The bars represent the average percentage of cells with permeabilized membranes, based on a minimum count of 100 cells per experiment. Each experiment was conducted independently in triplicate. The lines above the bars indicate the standard deviation from the three independent assays.

The results showed that rhamnolipid acts on the cytoplasmic membrane of *C. acnes* within 1 h of contact, with more than 65% of the cells stained red after this exposure period (Figure 9). Statistical analysis based on 300 cells from three independent experiments (n = 100 per experiment) revealed that, on average, 65.70% of the cells exhibited membrane damage (Figure 10). These findings support the efficacy of rhamnolipid in eliminating *C. acnes* and are consistent with the time-kill assay results, where more than half of the bacterial population was reduced within one hour of exposure.

The effect of rhamnolipid on bacterial cell division was also investigated using a mutant *Bacillus subtilis* strain expressing the Spo0J-GFP fusion protein. The mutant strain was exposed to rhamnolipid at its bactericidal concentration for 15 min and then examined under a fluorescence microscope using a green filter. In *B. subtilis*, the Spo0J protein (also known as ParB) plays a crucial role in chromosome segregation. Spo0J is a DNA-binding protein that specifically recognizes parS sites located near the origin of replication, forming dynamic foci that move within the cell during division, guiding chromosomes toward the cell poles [19,33,34,35].

When Spo0J is tagged with GFP, its movement and distribution within the cells can be visualized [19]. During complete bacterial cell division, Spo0J can be observed at the cell poles before the conclusion of binary fission [33]. The aim of this assay was to assess whether Spo0J-GFP remained localized at the cell poles after contact with rhamnolipid, thereby evaluating potential interference with bacterial cell division.

**Figure 11:**
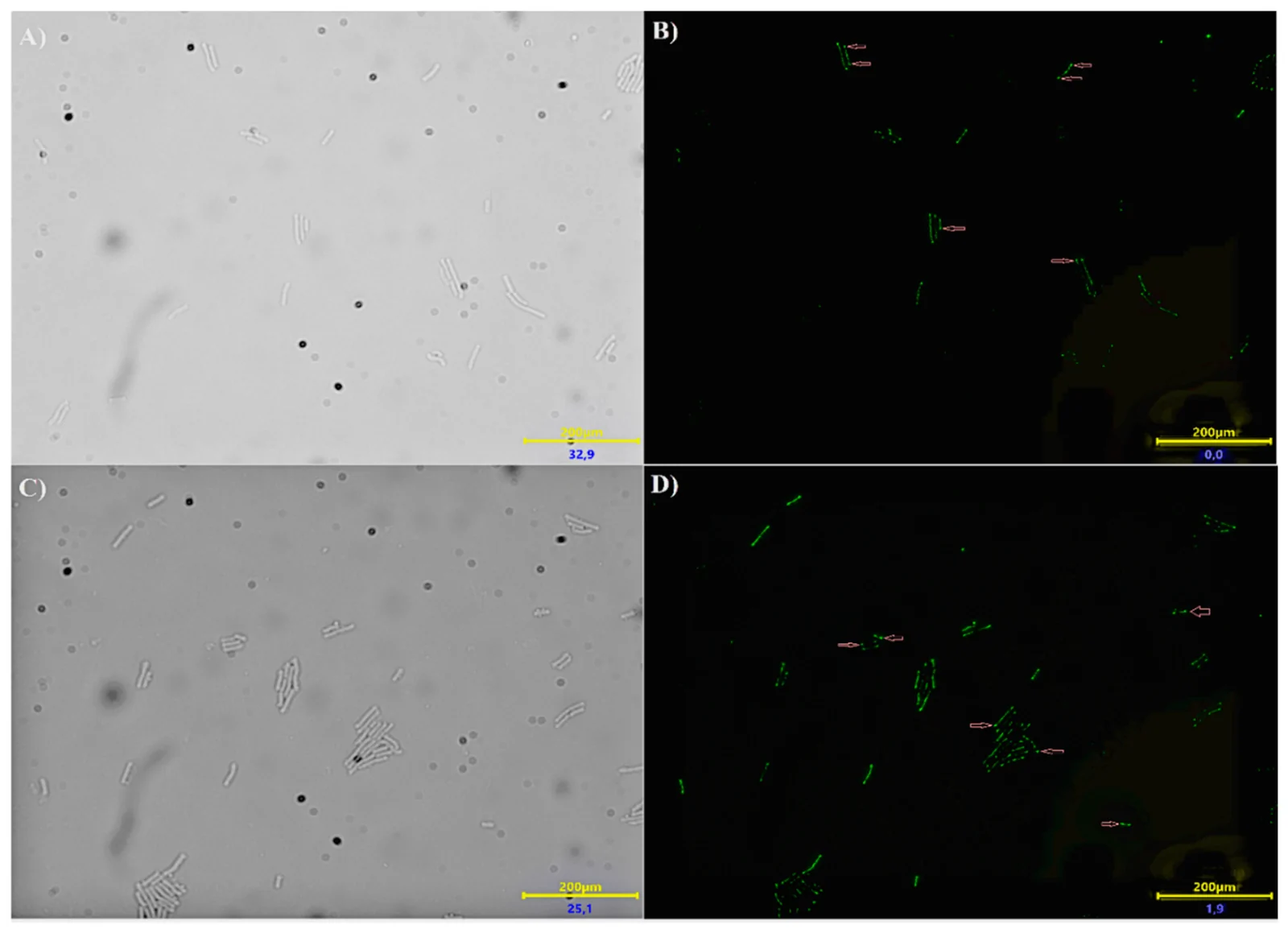
Fluorescence microscopy of the *B. subtilis* expressing Spo0J-GFP. The Spo0J-GFP at the poles, indicating successful division, is indicated by the red arrows. (**A**) Negative control with phase contrast (**B**) Negative control fluorescence with green filters. (**C**) cells after 15 min of contact with rhamnolipids with phase contrast. (**D**) cells after 15 min of contact with rhamnolipids with fluorescence with green filters. The scale bar corresponds to 200 μm; magnification of ×100.

The results showed that Spo0J-GFP localization at the cell poles was identical in both the control and rhamnolipid-treated groups. In a quantification of n = 100 cells observed per treatment, conducted in triplicate, only 6.98 ± 1.70% of aberrant cells or division-related issues were noted in the treatment group, compared to 6.01 ± 2.03% in the control. It is worth noting that a cellular error rate of up to 15% is generally considered normal for a bacterial cell population [17]. This finding suggests that rhamnolipid does not interfere with bacterial cell division (Figure 11). Therefore, based on fluorescence microscopy results, it can be stated that rhamnolipid acts primarily through surface contact with bacterial cells, causing damage to the cytoplasmic membrane. High-resolution images of all fluorescence microscopy assays are included in the Supplementary Material (Figures S1–S10).

### 3.5. Mechanical Properties of the Film

When developing bioactive films for treating skin diseases, it is essential to consider their behavior in the presence of exudates. This directly influences film adhesion, residence time at the site of action, the capacity for active ingredient release, mechanical resistance, and re-epithelialization. Therefore, the pharmacological performance of bioactive films depends on their hydrophilicity, thickness, liquid absorption capacity, and water vapor permeability [36,37]. The results of the mechanical characterizations of the films are shown in Table 5.

Thickness is a key parameter in skin delivery systems, as it can influence swelling behavior, water vapor permeability, and liquid absorption [37,38]. Among the three films analyzed, the sample containing the highest concentration of rhamnolipid exhibited greater thickness compared to the control and the low rhamnolipid concentration film, with a statistically significant difference (*p* < 0.05). This finding is associated with the higher concentration of the active compound in these samples.

Furthermore, the sample containing the highest rhamnolipid concentration was mechanically weaker (lower Ps values) than the other films, suggesting loose molecular interactions. Elongation at break (Eb%) did not differ significantly among the films. As discussed by Saraiva et al. (2023) [39], compared to other biological films, sodium alginate-based films have lower mechanical resistance, as also demonstrated in the present study. While Saraiva et al. [39] reported a rupture energy resistance of 7 N/mm for their sodium alginate film, the film produced in the present work exhibited a resistance of 4.7 N/mm (Table 5).

Despite this limitation, you can add other compounds that strengthen molecular interactions within the film matrix, such as calcium chloride or cellulose nanocrystals, which may improve the film’s mechanical properties [39].

The OEO 0.05% and TTO 0.2% membranes exhibited high Eb values of 159.2% and 160.6%, respectively, indicating highly ductile behavior under biaxial deformation. These results show that both membranes can withstand significant deformations before rupturing, a characteristic typical of materials with a greater capacity for plastic accommodation. Although both films show similar deformation, the TTO 0.2% film showed higher puncture resistance (0.183 MPa) and specific energy (0.147 MPa) than the OEO 0.05% film, which had values of 0.0548 MPa and 0.0416 MPa, respectively. This suggests that the TTO film combines high ductility with a greater capacity for energy absorption, resulting in overall better mechanical performance under puncture loading.

On the other hand, the OEO 0.5% film exhibited significantly different behavior. The Eb value was substantially lower (24.2%), evidencing stiffer behavior and lower deformation capacity before rupture. However, it presented the highest values of puncture resistance (0.625 MPa) and specific energy (0.629 MPa), indicating greater structural rigidity and greater resistance to the onset of failure. The elongation at break (Eb%) showed no significant difference between the films.

### 3.6. Liquid Absorption

Bacterial infections lead to the formation of exudates at the site of inflammation, which may also involve the development of new tissue. This newly formed tissue can react to external factors, including mechanical trauma during its growth. To prevent such trauma, it is important that the films used do not trigger clot formation at the application site.

The ability to keep the inflamed area moist is crucial for the success of film-based treatments. The film should not only be hydrophilic but also capable of absorbing exudates and retaining moisture in the affected area to maintain tissue integrity. Thus, evaluating the liquid absorption profile is vital for predicting the therapeutic effectiveness of the films. The liquid absorption values for the membranes can be found in Table 6.

### 3.7. Water Vapor Permeability (WVP)

In systems designed for skin application, both excessive fluid loss and excessive retention can hinder wound healing. Therefore, maintaining proper moisture balance is essential, making semipermeable films a desirable option for this purpose [38].

The evaluation of WVP is an important analysis in the development of films for topical use and can be influenced by several factors, such as the presence of cracks, porosity, composition, and thickness. The results obtained in this study (Table 6) indicated no statistically significant differences among the films (*p* < 0.05). This demonstrates that rhamnolipid incorporation did not alter the film’s permeability, preserving an important property for its intended application. When considering WVP, which takes into account the thickness of the material, a significant difference between the samples can be observed, revealing the intrinsic barrier behavior of each film. Film RL 800 µg/mL had the lowest WVP value, indicating better intrinsic water vapor barrier performance, even though it was the thinnest. This suggests a denser microstructure or lower vapor diffusivity through the polymer matrix.

On the other hand, film OEO 0.05% had the highest WVP value (4.86 × 10^−6^ g × m^−1^ × h^−1^ × Pa^−1^), approximately one order of magnitude higher than RL 800. Despite its greater thickness, its intrinsic permeability is higher, indicating that the increase in thickness did not compensate for the greater ease of vapor diffusion through the material.

The TTO 0.2% and CEO 0.5% films had intermediate WVP values (1.41 × 10^−6^ and 2.14 × 10^−6^, respectively). It is observed that, although OEC 0.5% is thicker than TTO 0.2%, its permeability is higher, suggesting structural or compositional differences that influence vapor diffusion.

### 3.8. In Vitro Bioadhesion

Bioadhesion refers to the capability of large molecules to stick to biological tissues. Bioadhesive systems provide several benefits as drug delivery platforms, such as longer retention time at the application site, increased surface contact area, reduced frequency of dosing, and ultimately improved patient adherence to treatment [40].

The bioadhesive force, or maximum adhesive strength, refers to the peak height of the stress–strain curve, representing the force required to detach the test sample from the substrate. The work of bioadhesion corresponds to the area under the stress–strain curve, which represents the energy needed to overcome the adhesive force.

The results show that the control films differed significantly from the rhamnolipid-containing films, indicating that incorporating the biosurfactant altered the formulation’s adhesive properties.

### 3.9. Antimicrobial Activity Assay of the Sodium Alginate Film

**Figure 12:**
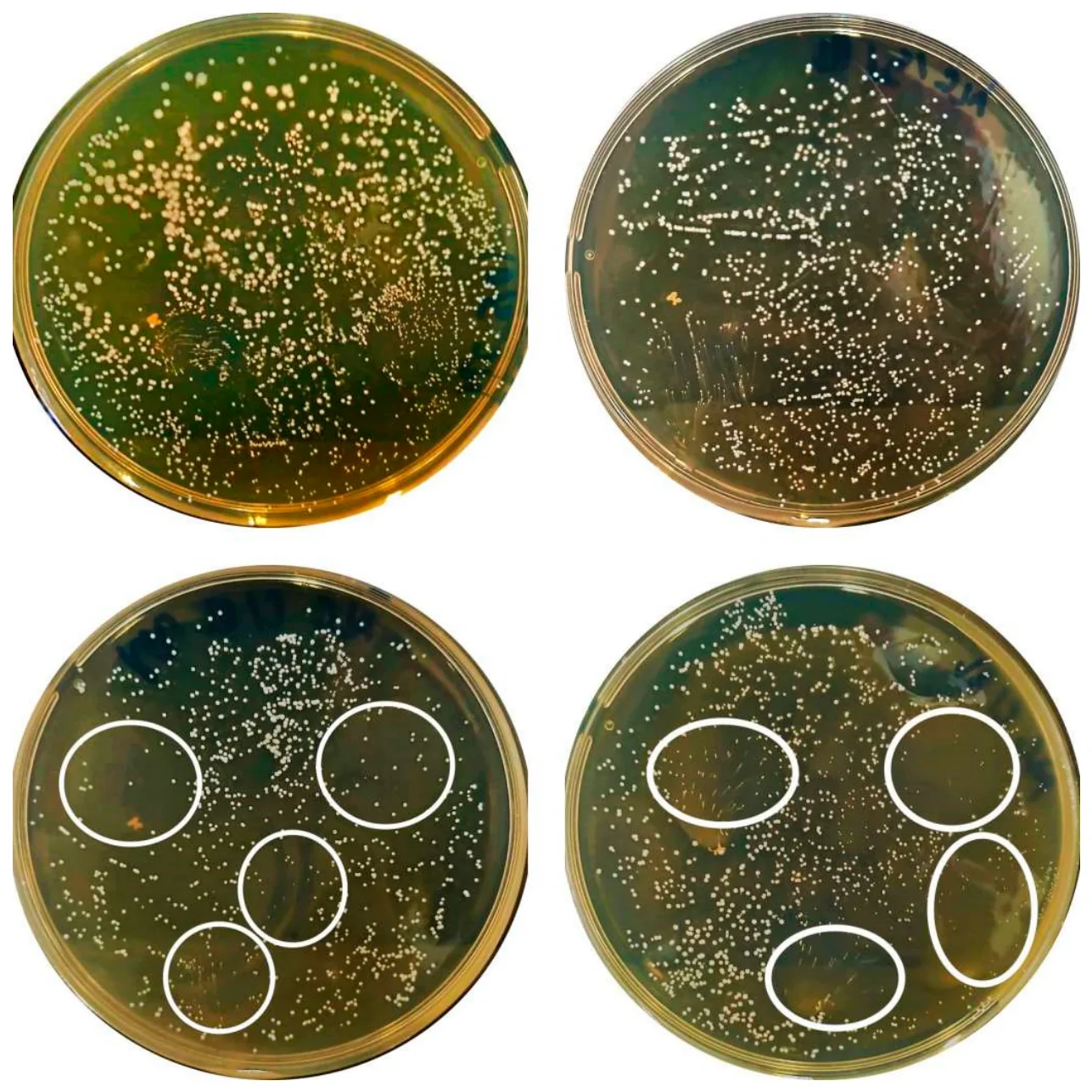
Photos of Petri dishes showing the lack of effect of the sodium alginate film on bacterial growth. The Petri dishes below demonstrate the impact of combining the sodium alginate film with rhamnolipids and essential oils on bacterial growth. The white circles delineate the area where a reduction in the growth of visible colonies occurred.

As the alginate discs gradually degrade and are absorbed into the medium, they are expected to release rhamnolipid and essential oils at the application site, which prevents microbial growth. The study demonstrated that, despite using an alginate-based formulation, the amount of rhamnolipid needed to inhibit the bacterial strain remained unchanged (Figure 12). Therefore, preparing a sodium alginate film to treat dermal infections is a viable option.

## 4. Discussion

This study confirms the established properties of biosurfactants and their potential as antimicrobial agents. Their primary mode of action occurs at the cell membrane. However, rhamnolipid exhibits limitations in its effectiveness, highlighting the need to combine multiple agents to enhance efficacy. Biosurfactants are derived from renewable raw materials and are considered safe for health, exhibiting low skin irritation with no evidence of sensitization or negative genotoxicity effects. These results were observed by More et al., (2026) [41].

In this context, essential oils, known for their well-documented antimicrobial properties, showed rapid, effective, and strong action against microorganisms. This effect was further enhanced when the oils were emulsified with rhamnolipid. The resulting emulsion forms a stable structure that facilitates mobility in culture media and helps retain the volatile components of the oils for a longer duration. This increases their effectiveness while reducing the concentration needed to achieve a bactericidal effect.

Finally, developing a sodium alginate-based film proved feasible. Combining the three compounds yields a resistant, semi-permeable structure that maintains antimicrobial activity, confirming early in vitro proof of concept.

## 5. Conclusions

This study demonstrated that rhamnolipid and essential oils represent a promising sustainable alternative for the treatment of dermal infections caused by *Cutibacterium acnes* and *Staphylococcus aureus*. Combining these naturally derived compounds increases antimicrobial activity, reducing the concentration needed to achieve bactericidal effects, enhancing biofilm inhibition, and accelerating bacterial elimination compared with the isolated compounds. Beyond their antimicrobial performance, the use of rhamnolipids and essential oils aligns with green chemistry principles by employing renewable bio-based molecules with low environmental impact and reduced dependence on conventional synthetic antimicrobials. The incorporation of these compounds into biodegradable sodium alginate films further reinforces the sustainability of the proposed system, providing a biocompatible platform capable of maintaining antimicrobial efficacy while minimizing the generation of persistent residues. Overall, the developed formulations demonstrate potential as environmentally friendly topical antimicrobial systems and support the growing transition toward sustainable pharmaceutical and healthcare products. Future studies should focus on in vivo evaluation, long-term stability, and scale-up production to enable the translation of these green technologies into clinical and commercial applications.

## Figures and Tables

**Table 1:** Compounds and their respective MBCs against *Cutibacterium acnes*.

Compound	Concentration
RHL	800 µg/mL
OEO	0.05% (*v*/*v*)
TTO	0.2% (*v*/*v*)
CEO	0.5% (*v*/*v*)

**Table 2:** Compounds and their respective MBCs against *Staphylococcus aureus*.

Compound	Concentration
RHL	2500 µg/mL
OEO	0.07% (*v*/*v*)
TTO	0.3% (*v*/*v*)
CEO	0.5% (*v*/*v*)

**Table 3:** Emulsion and its respective MBCs against *Cutibacterium acnes*.

Compound	Concentration
RHL + OEO	500 µg/mL + 0.03%
RHL + TTO	500 µg/mL + 0.1%
RHL + CEO	500 µg/mL + 0.3%

**Table 4:** Emulsion and its respective MBCs against *Staphylococcus aureus*.

Compound	Concentration
RHL + OEO	1500 µg/mL + 0.05%
RHL + TTO	1500 µg/mL + 0.1%
RHL + CEO	1500 µg/mL + 0.3%

**Table 5:** Thickness, elongation at breaking (Eb%), puncture strength drilling resistance (Ps), and energy at breaking (Eb) of SA films.

Sample	Thickness (mm)	Eb (%)	Ps (MPa)	Ep (N.s^−1^/mm^3^)
Control	0.026 ± 0.009 ^a^	21.31 ± 3.35 ^a^	56.748 ± 7.620 ^b^	4.782 ± 0.693 ^b^
RL 100 µg/mL	0.035 ± 0.007 ^a^	18.729 ± 0.572 ^a^	45.614 ± 10.587 ^a^	2.126 ± 1.169 ^a^
RL 400 µg/mL	0.047 ± 0.006 ^b^	25.605 ± 3.373 ^a^	38.504 ± 11.113 ^a^	7.278 ^a^ ± 3.744 ^c^
RL 800 µg/mL	0.043 ± 0.008 ^a^	32.31 ± 4.25 ^a^	6.432 ± 0.620 ^b^	2.782 ± 0.923 ^c^
OEO 0.05%	0.41 ± 0.08 ^b^	159.2 ± 0.3 ^b^	0.055 ± 10.587 ^a^	0.042 ± 0.069 ^a^
TTO 0.2%	0.12 ± 0.09 ^a^	160.6 ± 3.3 ^b^	0.18 ± 0.13 ^a^	0.147 ^a^ ± 0.744 ^b^
CEO 0.5%	0.16 ± 0.05 ^a^	24.2 ± 2.3 ^a^	0.62 ± 0.11 ^a^	0.63 ^a^ ± 0.44 ^b^

^a^, ^b^ and ^c^ denote a significant difference between analyses determined using Tukey’s multiple comparison test (*p* < 0.05).

**Table 6:** Water vapor permeability (WVP), mucoadhesive force (F_MA_) and mucoadhesion work (W_MA_). Results are expressed as mean ± standard deviation (SD).

Sample	WVP (×10^−5^ g mm m^−2^ h^−1^ Pa^−1^)	Liquid Uptake Ability (%)	F_MA_ (N)	W _MA_ (N.s)
Film control	6.06 ± 1.01 ^a^	11.12 ± 1.26 ^a^	1.0611 ± 0.6516 ^a^	0.362 ± 0.244 ^b^
RL 100 µg/mL	6.60 ± 0.92 ^a^	15.02 ± 3.22 ^a^	0.287 ± 0.326 ^b^	0.091 ± 0.138 ^a^
RL 400 µg/mL	6.12 ± 1.23 ^a^	10.60 ± 6.01 ^a^	0.359 ± 0.302 ^b^	0.143 ± 0.135 ^a^
OEO 0.05%	4.86 × 10^−6 b^	13.82 ± 0.23 ^a^	0.294 ± 0.132 ^b^	0.182 ± 0.138 ^a^
TTO 0.2%	1.41 × 10^−6 a^	13.94 ± 1.53 ^a^	0.123 ± 0.012 ^b^	0.183 ± 0.118 ^a^
CEO 0.5%	2.4 × 10^−6 a^	12.60 ± 2.31 ^a^	0.421 ± 0.232 ^b^	0.112 ± 0.012 ^a^

^a^ and ^b^ denote a significant difference between analyses determined using Tukey’s multiple comparison test (*p* < 0.05).

## Data Availability

The original contributions presented in this study are included in the article. Further inquiries can be directed to the corresponding authors.

## Supplementary Materials

The following supporting information can be downloaded at: https://www.mdpi.com/article/10.3390/microorganisms14102254/s1.
