# Supplementary material for: Rhamnolipids and Essential Oils: A New Approach to Managing Bacteria Associated with Skin Diseases
Source: Microorganisms. 2026 Oct 4;14(10):2254. doi: 10.3390/microorganisms14102254 (PMC13707553; doi:10.3390/microorganisms14102254)
Supplement: Supplementary file 1 [file microorganisms-14-02254-s001.zip › microorganisms-4542789-supplementary.pdf]

Synergistic Potential of Rhamnolipid and Essential Oils Against *Cutibacterium acnes* and *Staphylococcus aureus*: Development of a Sodium Alginate-Based Antimicrobial Film

****Supplementary Material****

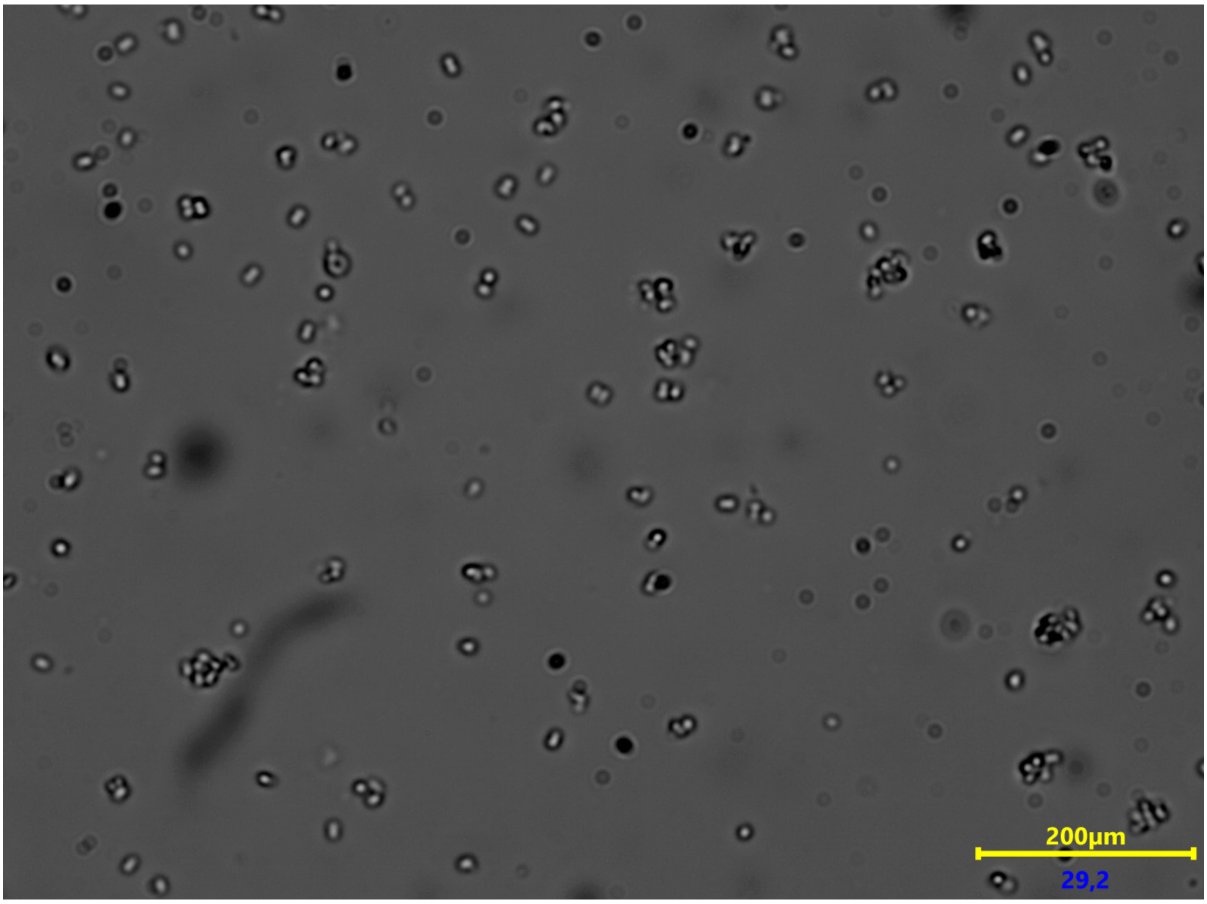

Supplementary Figure S1. Microscopy fluorescence of *C. acnes*. Original photo of the phase contrast of the negative control. Scale bar 200 µm; magnification of 100×.

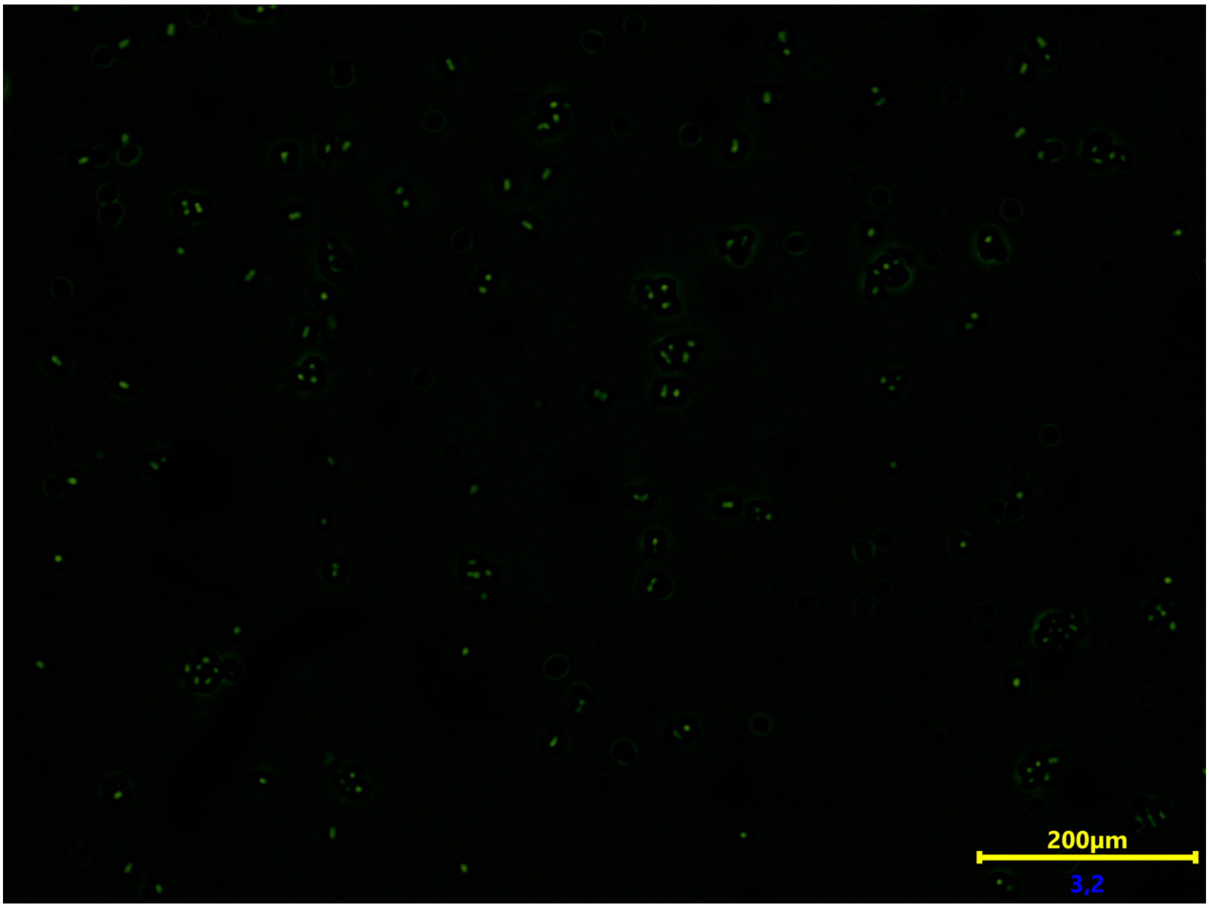

Supplementary Figure S2. Microscopy fluorescence of *C. acnes*. Original photo of the negative control at fluorescence phase with green and red filters. Scale bar 200 µm; magnification of 100×.

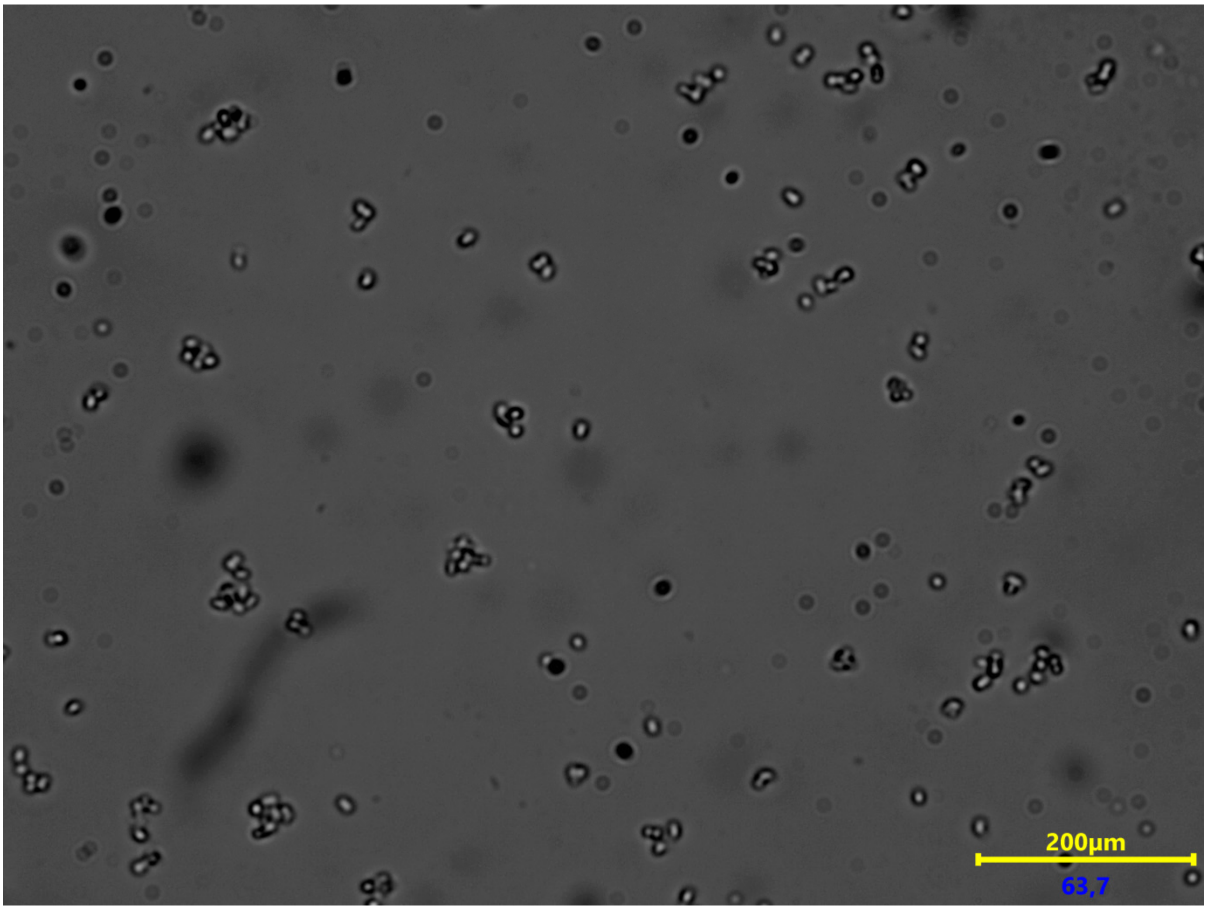

Supplementary Figure S3. Microscopy fluorescence of *C. acnes*. Original photo of the phase contrast of the positive control. Scale bar 200 µm; magnification of 100×.

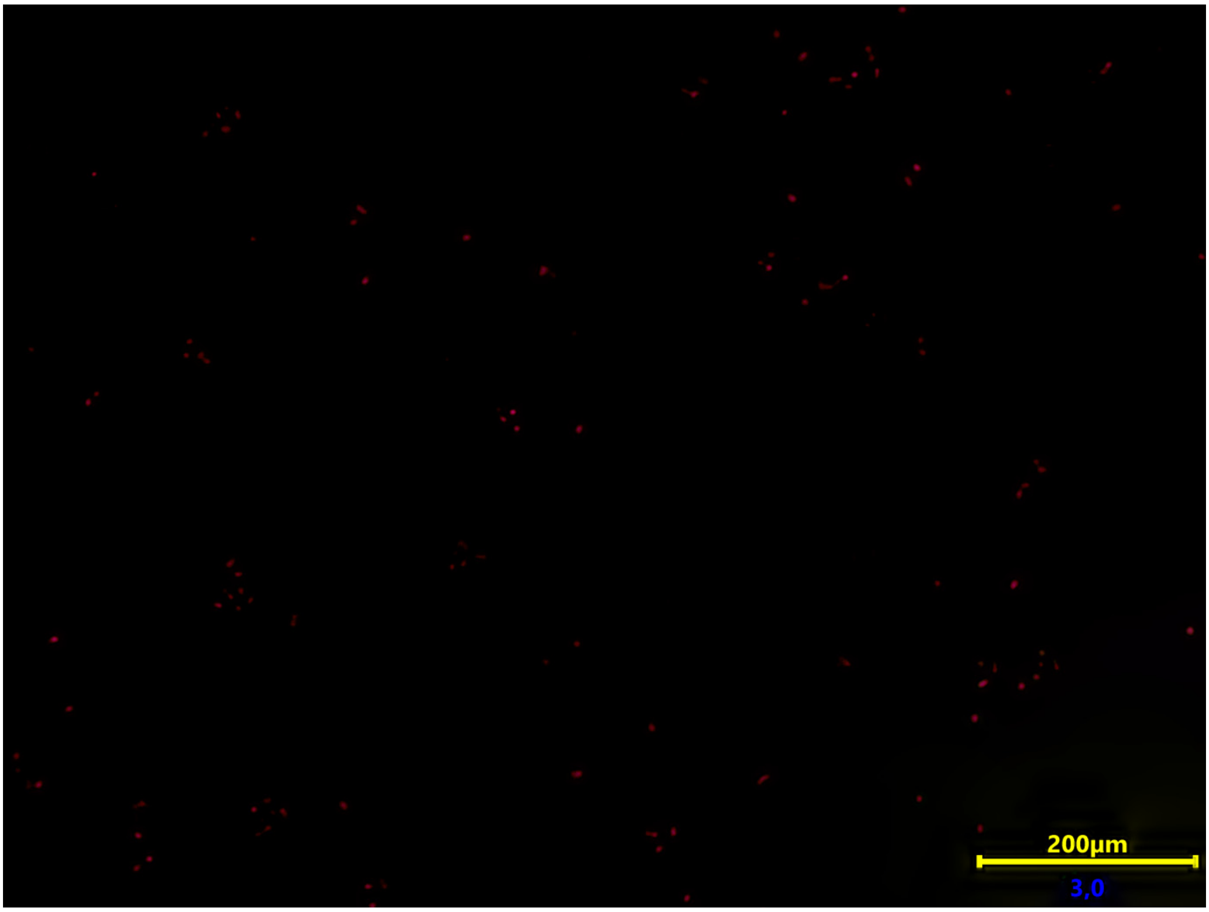

Supplementary Figure S4. Microscopy fluorescence of *C. acnes*. Original photo of the positive control at fluorescence phase with green and red filters. Scale bar 200 µm; magnification of 100×.

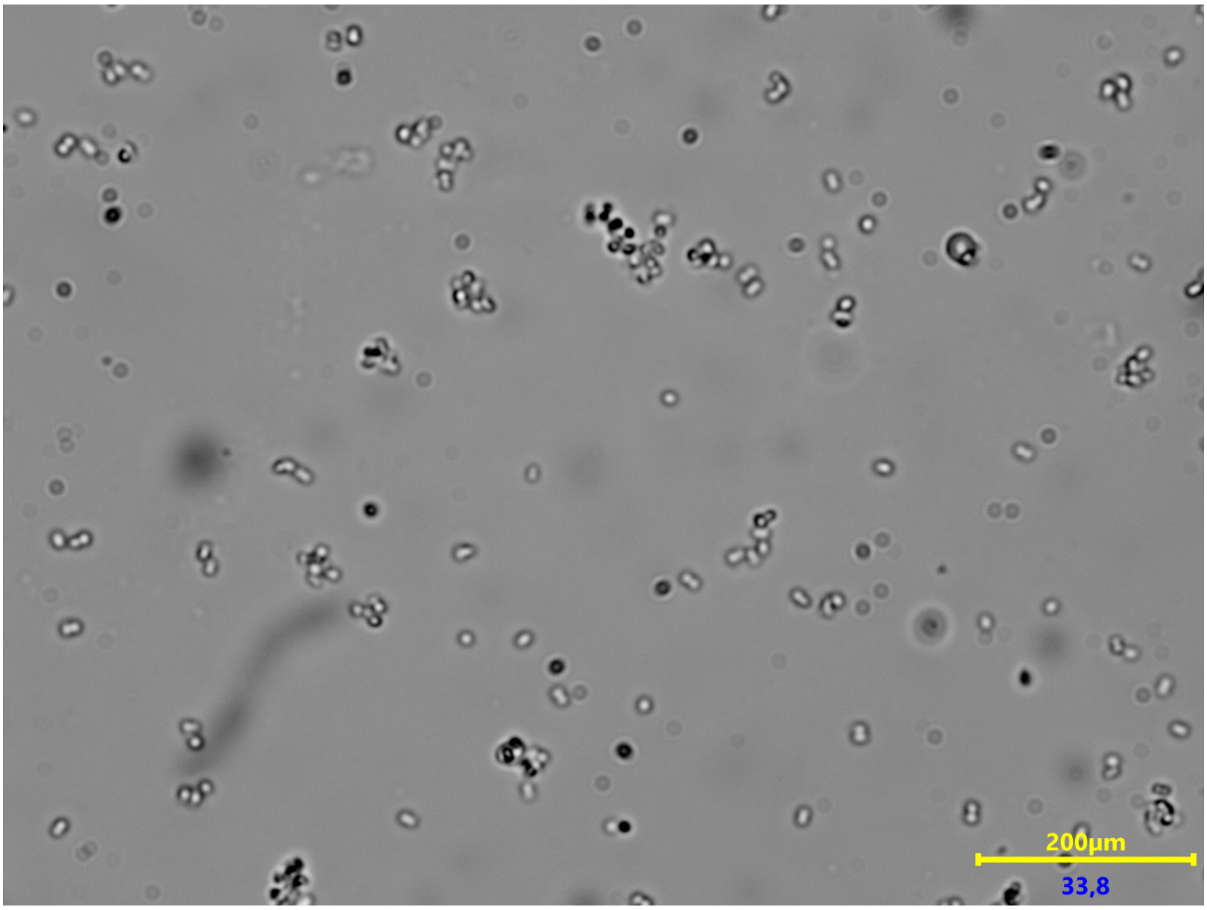

Supplementary Figure S5. Microscopy fluorescence of *C. acnes* after contact with 800 µg/mL of rhamnolipids. Original photo of the phase contrast of the *C. acnes* cells after 30 min of contact with rhamnolipids. Scale bar 200 µm; magnification of 100×.

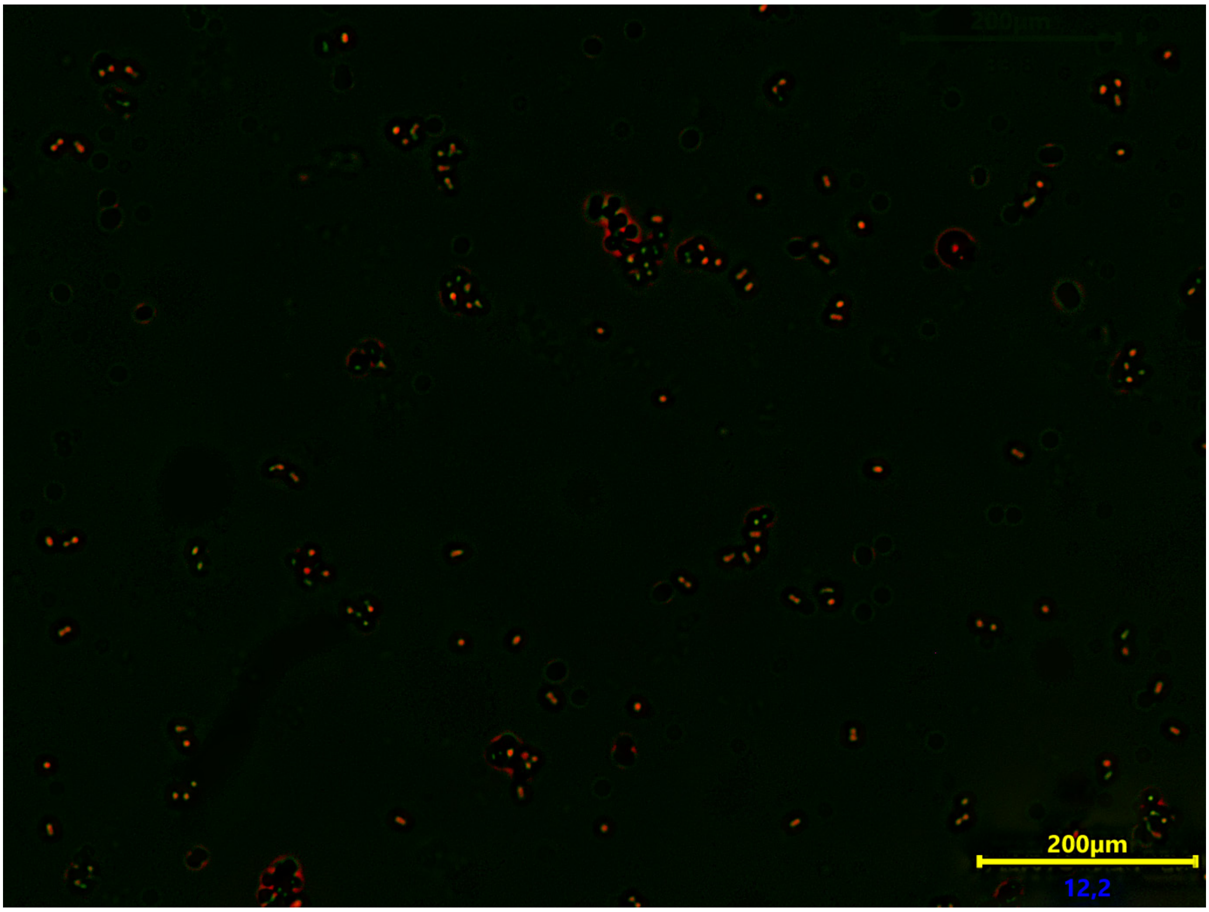

Supplementary Figure S6. Microscopy fluorescence of *C. acnes* after contact with 800 µg/mL of rhamnolipids. Original photo of the of the *C. acnes* cells after 30 min of contact with rhamnolipids at fluorescence phase with green and red filters. Scale bar 200 µm; magnification of 100×.

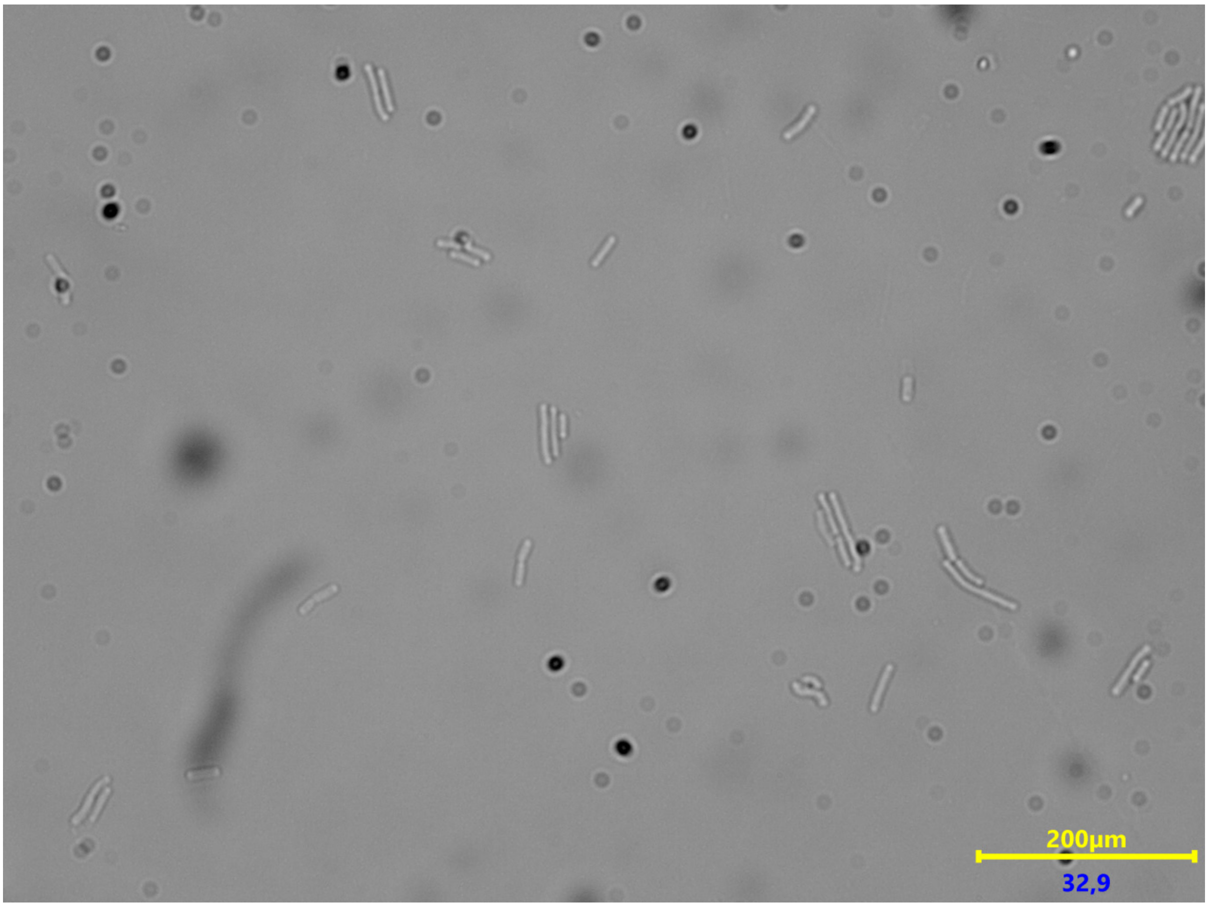

Supplementary Figure S7. Fluorescence microscope of the *B. subtilis* expressing Spo0J-GFP. Original photo of the phase contrast of the negative control. The scale bar corresponds to 200 µm; magnification of ×100.

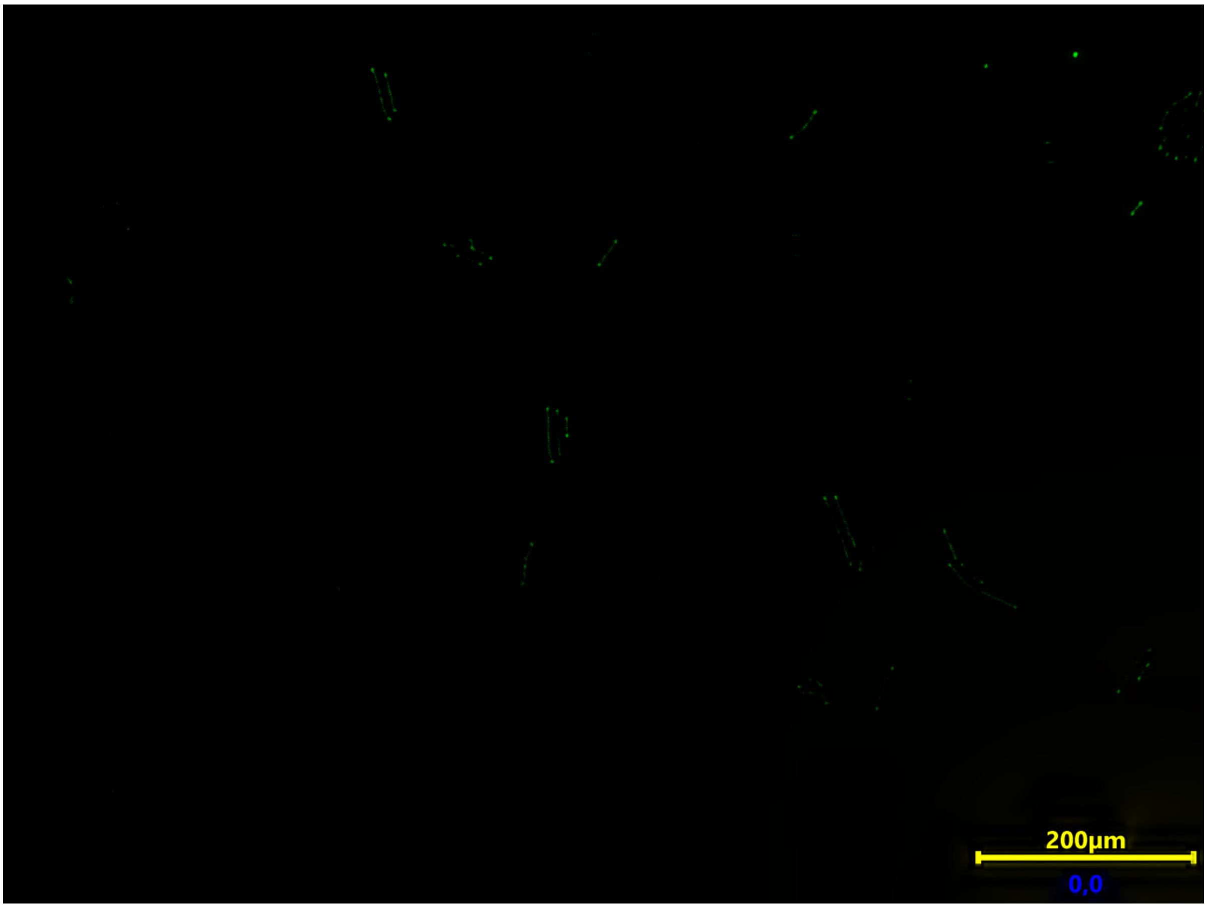

Supplementary Figure S8. Fluorescence microscope of the *B. subtilis* expressing Spo0J-GFP. Original photo of the negative control at fluorescence phase with green filter. The Spo0J-GFP at the poles, indicating a successful division. The scale bar corresponds to 200 μm; magnification of ×100.

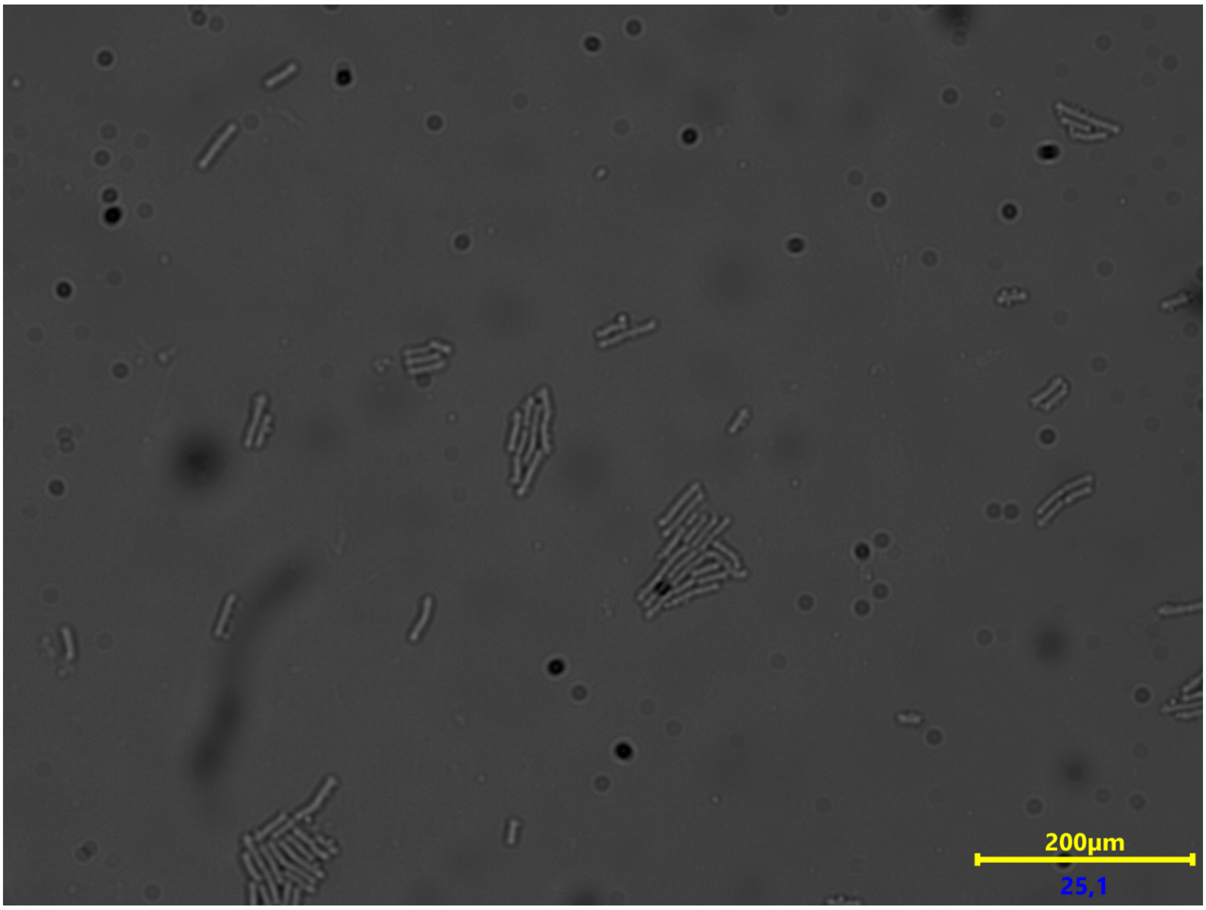

Supplementary Figure S9. Fluorescence microscope of the *B. subtilis* expressing Spo0J-GFP. Original photo of the phase contrast of the mutant *B. subtilis* cells after 15 min of contact with rhamnolipids. The scale bar corresponds to 200 µm; magnification of ×100.

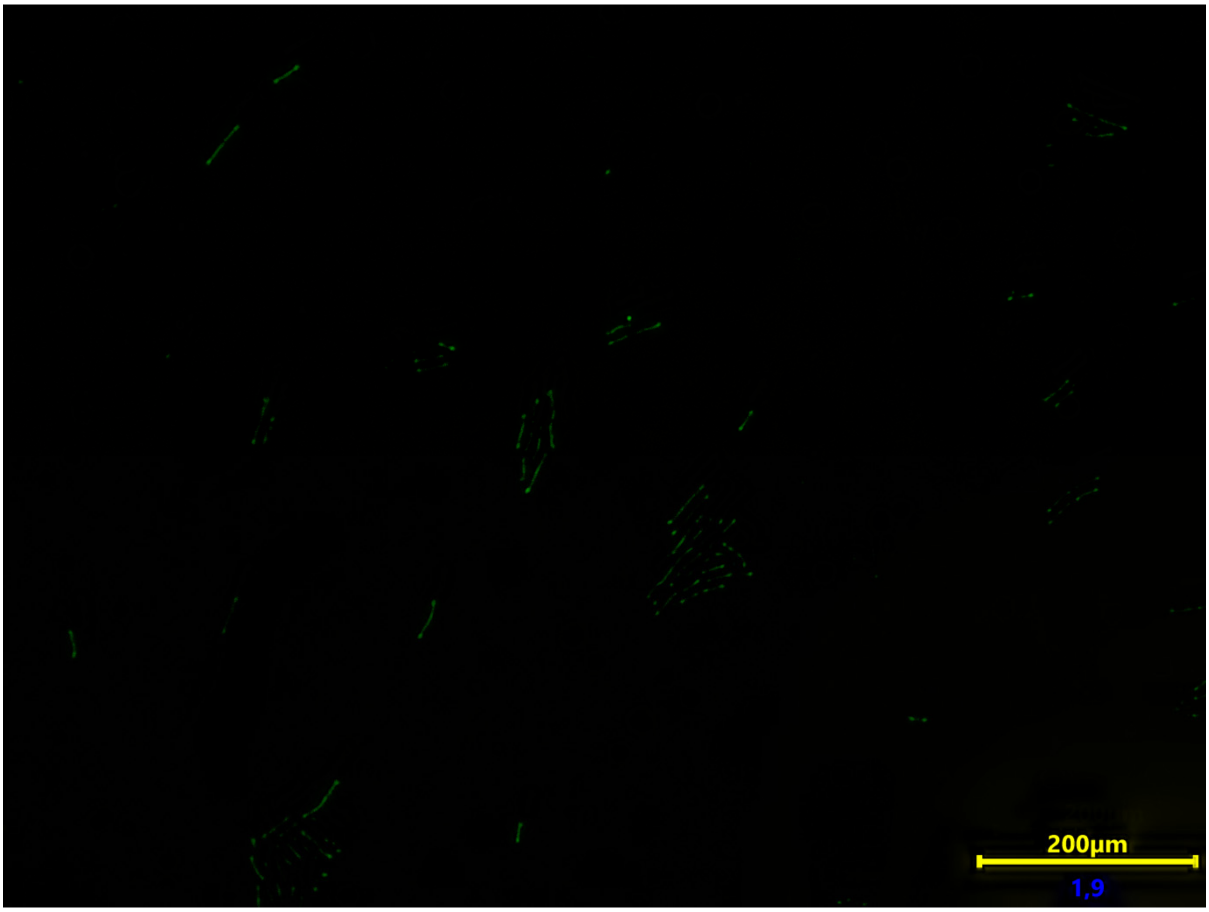

Supplementary Figure S10. Fluorescence microscope of the *B. subtilis* expressing Spo0J-GFP. Original photo of the mutant *B. subtilis* cells after 15 min of contact with rhamnolipids at fluorescence phase with green filter. The Spo0J-GFP at the poles, indicating a successful division. The scale bar corresponds to 200 µm; magnification of ×100.
